# The peroxisomal AAA-ATPase Pex1/Pex6 unfolds substrates by processive threading

**DOI:** 10.1038/s41467-017-02474-4

**Published:** 2018-01-10

**Authors:** Brooke M. Gardner, Dominic T. Castanzo, Saikat Chowdhury, Goran Stjepanovic, Matthew S. Stefely, James H. Hurley, Gabriel C. Lander, Andreas Martin

**Affiliations:** 1 Department of Molecular and Cell Biology, University of California, Berkeley, Berkeley, CA 94720 USA; 2 Department of Integrative Structural and Computational Biology, The Scripps Research Institute, 10550 North Torrey Pines Road, La Jolla, CA 92037 USA; 3 Molecular Biophysics and Integrated Bioimaging Division, Lawrence Berkeley National Laboratory, Berkeley, CA 94720 USA; 4 California Institute for Quantitative Biosciences, University of California, Berkeley, Berkeley, CA 94720 USA; 5 Howard Hughes Medical Institute, University of California, Berkeley, Berkeley, CA 94720 USA

## Abstract

Pex1 and Pex6 form a heterohexameric motor essential for peroxisome biogenesis and function, and mutations in these AAA-ATPases cause most peroxisome-biogenesis disorders in humans. The tail-anchored protein Pex15 recruits Pex1/Pex6 to the peroxisomal membrane, where it performs an unknown function required for matrix-protein import. Here we determine that Pex1/Pex6 from *S*. *cerevisiae* is a protein translocase that unfolds Pex15 in a pore-loop-dependent and ATP-hydrolysis-dependent manner. Our structural studies of Pex15 in isolation and in complex with Pex1/Pex6 illustrate that Pex15 binds the N-terminal domains of Pex6, before its C-terminal disordered region engages with the pore loops of the motor, which then processively threads Pex15 through the central pore. Furthermore, Pex15 directly binds the cargo receptor Pex5, linking Pex1/Pex6 to other components of the peroxisomal import machinery. Our results thus support a role of Pex1/Pex6 in mechanical unfolding of peroxins or their extraction from the peroxisomal membrane during matrix-protein import.

## Abstract (web summary)

Pex1 and Pex6 form a heterohexameric Type-2 AAA-ATPase motor whose function in peroxisomal matrix-protein import is still debated. Here, the authors combine structural, biochemical, and cell-biological approaches to show that Pex1/Pex6 is a protein unfoldase, which supports a role in mechanical unfolding of peroxin proteins.

## Introduction

Peroxisomes are membrane-bound organelles that perform specialized metabolic reactions, including the β-oxidation of very long chain fatty acids^1^. The function, size, and number of peroxisomes can be adjusted by eukaryotic cells according to metabolic needs^2^. Peroxisomes are formed by either division of existing peroxisomes or by de novo biogenesis, a process that depends on ~35 dedicated peroxin (Pex) proteins^3^. Disruption of peroxisome function through mutations of Pex genes in humans causes a spectrum of developmental disorders called peroxisome biogenesis disorders (PBDs). The severity of these disorders depends on how the mutation affects the formation of functional peroxisomes^4^.

Current models of de novo peroxisome biogenesis posit that the peroxisomal membrane proteins traffic through the endoplasmic reticulum or mitochondria, and subsequently bud into pre-peroxisomal vesicles that fuse to form an empty membrane-bound compartment, the “peroxisome ghost”^5,6^. Peroxisomal matrix proteins are made in the cytosol and targeted to peroxisomes by one of two peroxisomal targeting signals: PTS1, a C-terminal tripeptide, or PTS2, an N-terminal nonapeptide^7,8^. Distinct receptor proteins recognize the targeting signals (Pex5 for PTS1, Pex7 for PTS2), shuttle the matrix proteins to the peroxisomal membrane, and then interact with the docking complex Pex13/14^9–11^. By an unknown mechanism, the cargo receptors mediate the import of the fully folded matrix protein into the peroxisome. During this import, the cargo receptors assume a protease-protected state, likely embedded in the peroxisomal membrane^12,13^. A transmembrane complex of RING domain-containing E3 ubiquitin ligases, Pex2/10/12, then ubiquitinates an N-terminal cysteine of the cargo receptors and thereby primes them for membrane extraction and subsequent rounds of import^14–17^.

Pex1 and Pex6 form a single, heterohexameric Type-2 AAA-ATPase motor with an architecture similar to Cdc48/p97 and NSF, but with alternating subunits^18–20^. Akin to the roles of NSF in the recycling of SNARE proteins and Cdc48/p97 in ER-associated degradation (ERAD), Pex1/Pex6 has been proposed to have roles both in peroxisome vesicle fusion^5,21^ and in extraction of the ubiquitinated cargo receptors from peroxisomal membranes^17,22–24^. Closer examination of cells lacking functional Pex1 revealed empty peroxisome membrane compartments^25^ and continued delivery of peroxisome membrane proteins^26^, indicating that Pex1/Pex6’s primary role is in peroxisomal matrix-protein import. Without functional Pex1 or Pex6, ubiquitinated Pex5 accumulates on peroxisomes, which are then targeted for specific autophagy^22,27,28^, suggesting that Pex1/Pex6 is also important for peroxisome stability. While inhibiting autophagy can stabilize peroxisomes and allow peroxisomal matrix-protein import in cells with hypomorphic Pex1, it could not recover peroxisome function in Pex1-null cells^28^. It therefore appears that Pex1/Pex6 has a primary role in peroxisomal matrix-protein import, whose impairment leads to peroxisome-specific autophagy.

Like other Type-2 AAA-ATPases, the Pex1/Pex6 motor contains two stacked rings of ATPase domains, termed D1 and D2, which are preceded by N-terminal domains that are typically involved in binding of substrates or cofactors^29^. Pex1 and Pex6 each have two N-terminal domains that are structurally related to the singular N-terminal domains on Cdc48/p97 and NSF^20,30^. The nucleotide-binding pockets of AAA-ATPases are located at the interfaces between neighboring subunits, with the Walker A (WA) and Walker B (WB) motifs contributed by one subunit, and the arginine-finger/box VII motif provided by the clockwise-next neighbor. In the yeast *Saccharomyces cerevisiae*, all Pex1/Pex6 ATP hydrolysis derives from the C-terminal D2 ring, which contains active ATPase sites at the Pex1–Pex6 and the Pex6–Pex1 interfaces^18,19^. Mutational analyses of these D2 sites revealed that the ATPase activity of Pex1 is strongly coordinated with that of Pex6^18^.

Hexameric AAA-ATPases usually employ one of two main mechanisms to couple ATP binding and hydrolysis with the mechanical disassembly of protein substrates. Protein translocases-like Cdc48/p97, Vps4, and Clp’s^31–34^ utilize ATP-hydrolysis-driven conformational changes in their AAA-motor domains to thread substrates through the central pore of the hexamer. In these translocases, pore loops that contain a conserved aromatic-hydrophobic dipeptide motif engage a disordered region of the substrate, and apply unfolding force to structured domains by attempting to pull them through the narrow central pore^33^. Non-processive protein remodelers such as NSF also contain these conserved pore loops, but use them to hold and stabilize the substrate above the AAA ring, while large nucleotide-dependent conformational changes in their N-terminal domains facilitate substrate unfolding or rearrangement^35,36^. It had previously been observed that yeast growth on oleic acid, which requires peroxisomal β-oxidation, depends on intact pore loops in the Pex1 and Pex6 D2 domains^19^. However, it is unclear whether Pex1/Pex6 functions as a protein unfoldase that threads its substrates or a remodeler that exerts force through its N-terminal domains. Pex1/Pex6’s proposed function in pre-peroxisomal vesicle fusion^5,21^ resembles the activity of NSF, while its potential role in cargo-receptor extraction resembles the threading mechanism of Cdc48/p97 in ERAD. The membrane-embedded, ubiquitinated cargo receptors have long been considered substrates of Pex1/Pex6, because cells lacking functional Pex1/Pex6 accumulate ubiquitinated Pex5 on the peroxisomal membranes, and exogenously added Pex1/Pex6 activity prompts the release of Pex5 from peroxisomes in vitro^17^. However, there is no evidence of Pex1/Pex6 robustly binding or processing Pex5, and the lack of any known substrate has so far prevented further investigations into Pex1/Pex6’s mechanism for substrate processing.

The function of Pex1/Pex6 depends on the tail-anchored membrane protein Pex15 in yeast or Pex15’s homologs Pex26 in humans and APEM9 in plants^37–40^. Despite low sequence identity, Pex15, Pex26, and APEM9 appear to have a similar function of recruiting Pex1/Pex6 to the peroxisomal membrane. Deletions of Pex15 closely resemble the phenotype of Pex1 and Pex6 deletions, leading to the accumulation of ubiquitinated Pex5 at the peroxisome membrane and to increased peroxisome-specific autophagy^27^. Genetic and biochemical evidence suggests that Pex15 uses its N-terminus to directly bind to Pex6^41,42^, which appears to be a common mechanism amongst homologs^43,44^. We recently found that the cytosolic domain of Pex15 inhibits the basal ATPase activity of Pex1/Pex6^18^, and Grimm et al. proposed that Pex15 binding to Pex1/Pex6 depends on the nucleotide state of Pex6^42^. These observations suggested that Pex15 was not just a passive binding partner, but may function as a regulator of Pex1/Pex6 ATPase activity.

Here we show that Pex15 inhibits the ATPase activity of Pex1/Pex6 because its cytosolic portion is recognized as a substrate, engaged by the pore loops, and completely unfolded by the AAA motor. Using a combination of X-ray crystallography, negative-stain electron microscopy (EM), and hydrogen deuterium exchange with mass spectrometry (HDX-MS), we find that Pex15 initially binds Pex1/Pex6 at the N1/N2 domain interface of Pex6, which positions the disordered C-terminal region of Pex15 for engagement by the D2 pore loops. Pex1/Pex6 then unfolds Pex15 and any N-terminally fused proteins by processive threading through the central pore in an ATP-hydrolysis-dependent manner. We found that a pore-loop mutant of Pex1/Pex6 is temperature sensitive in both our Pex15-unfolding assays in vitro and in peroxisomal matrix-protein import in vivo, suggesting a corresponding mechanism of action. While the identity of in vivo substrates remains unclear, Pex1/Pex6’s identified function as a protein unfoldase is consistent with a role in extraction of Pex proteins from the peroxisomal membrane.

## Results

### Pex15 inhibits Pex1/Pex6 in a pore-loop-dependent manner

**Fig. 1:**
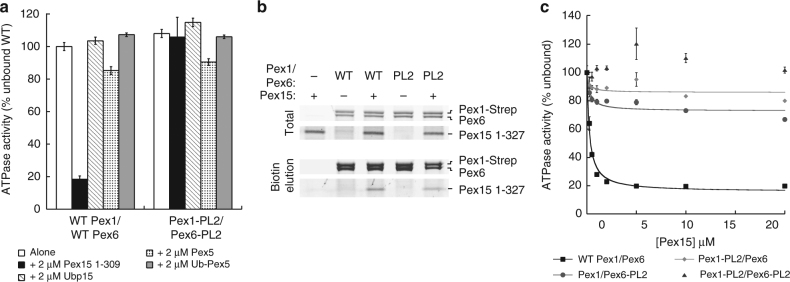
Pex15 inhibits the ATPase activity of Pex1/Pex6 in a pore-loop-dependent manner. **a** ATPase activity of wild-type (WT) and D2 pore-loop (PL2) mutant Pex1/Pex6 alone and in the presence of Pex15, Ubp15, Pex5, or a linear ubiquitin-Pex5 fusion (mean and s.d. for *n* = 3 technical replicates). The D2 pore loop mutations are Pex1-F771A and Pex6-Y805A. **b** Pex15’s cytosolic domain binds both wild-type Pex1/Pex6 and the Pex1/Pex6 D2 pore-loop mutant. **c** Pore-loop mutations in either Pex1 or Pex6 reduce the inhibitory effect of Pex15. ATPase data shown are the mean±s.d. for *n* = 3 technical replicates

We previously observed that the cytosolic domain of Pex15 inhibits the basal ATPase activity of the recombinant Pex1/Pex6 motor in vitro^18^. Changes in the basal rate of other AAA-ATPases can be caused either by binding of a cofactor or by substrate processing^31,45,46^. Since both main mechanisms of substrate processing by AAA motors, threading and external remodeling, involve the central pore loops, we compared the ATPase activities of wild type and D2 pore-loop mutated Pex1/Pex6 in the presence and absence of Pex15. Interestingly, mutation of the aromatic pore-loop residues to alanine in the D2 domains of both Pex1 (F771A) and Pex6 (Y805A) eliminated the ATPase inhibition by Pex15, but did not significantly affect the basal ATP-hydrolysis rate (Fig. 1a). Successful pull-down binding assays ruled out that this lack of ATPase response is due to a Pex15-binding defect (Fig. 1b), and titration experiments confirmed that Pex15 concentrations of up to 20 μM had no effect on the ATP-hydrolysis activity of pore-loop mutated Pex1/Pex6 (Fig. 1c). When pore loops are mutated in only Pex1 or Pex6, however, Pex15 still inhibits ATPase activity, albeit much more weakly (Fig. 1c). Since Pex1/Pex6 has also been proposed to act on the ubiquitinated cargo receptor Pex5^14,15,17^ and bind the de-ubiquitinating enzyme Ubp15^47^, we tested the effects of recombinant Ubp15, Pex5, and a linear ubiquitin–Pex5 fusion on the motor’s ATPase activity as well, but observed minimal changes that did not depend on the D2 pore loops (Fig. 1a). The pore-loop dependence of Pex1/Pex6’s ATPase inhibition by Pex15 thus suggests that the cytosolic portion of Pex15 is recognized and processed as a substrate.

### Pex15 structure and interaction with Pex1/Pex6

**Fig. 2:**
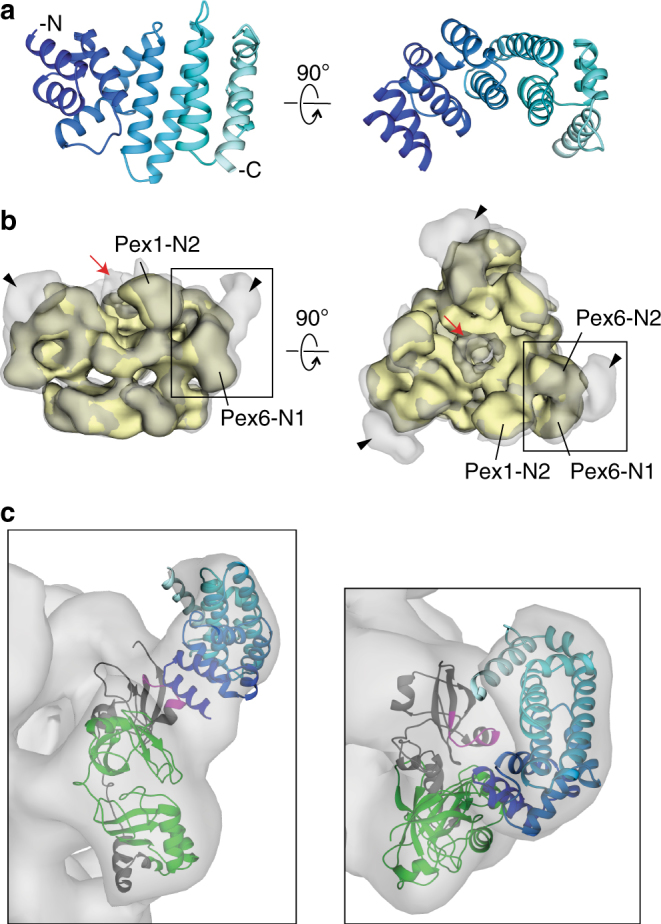
Structure of the Pex15 core domain and its interaction with Pex1/Pex6. **a** A ribbon diagram of Pex15’s core domain (aa 43–253) is shown in a blue spectrum with the darkest blue at the N terminus (see also Supplementary Figure 1C). **b** 3D reconstructions from negative-stain EM of wild-type Pex1/Pex6 alone (yellow, EMD-6254) and in complex with the cytosolic domain of Pex15 (aa 1–327) at 23.2 Å resolution (gray, EMD-7005), showing additional density above the Pex6 N-terminal domains (arrowhead) and the central pore (red arrow) (see also Supplementary Figure 2). **c** Close-up views of the rigid-body fit of the Pex15 crystal structure (blue spectrum as in **a**) in the additional density above the Pex6 N-terminal domains. An atomic model of the Pex6 N-terminal domains^20^ in ribbon representation is colored gray, with the regions protected by Pex15 in the HDX-MS experiments in green (the N1 domain) and magenta (Pex6 aa 241–250) (see also Supplementary Figures 3, 4 and Supplementary Table 2)

Pex15 and its homologs are tail-anchored proteins with poor sequence conservation and no known structure, yet they share a common function in recruiting the Pex1/Pex6 AAA-ATPase motor to the peroxisome membrane. Using limited proteolysis on the cytosolic domain of yeast Pex15, we determined that Pex15 consists of a stable core region (aa 43–253) with disordered N-terminal and C-terminal tails (Supplementary Figure 1). We confirmed this result using hydrogen deuterium exchange coupled with mass spectrometry (HDX-MS) (Supplementary Figure 1). HDX-MS monitors the exchange of a protein’s backbone amide hydrogens for deuterium from the solvent, which depends on solvent accessibility and hydrogen bonding and reports on the folded state of the protein^48,49^. HDX-MS analysis of Pex15 showed 72% peptide coverage and confirmed that peptides at the N and C termini were quickly deuterated in contrast to those within the region protected from limited proteolysis. Without these disordered regions, the 25 kDa core was readily amenable to crystallization, and we solved its structure at 1.55 Å resolution (Fig. 2a, Supplementary Figure 1c, Supplementary Table 1). The core consists of 12 α-helices that fold into a compact, curved structure stabilized by a network of hydrophobic interactions. Structure predictions of Pex26 by JPred4 also indicated an entirely α-helical fold^50^, suggesting that despite its highly diverged primary sequence, this homolog shares a common structure with Pex15.

Previous EM studies of Pex1/Pex6 in the presence of different nucleotides did not reveal any large conformational changes of the N-terminal domains^18,19^ similar to those observed for protein remodelers such as NSF. However, these studies could not rule out that alternative conformations are only transiently adopted and potentially stabilized by binding partners or substrates. To determine if Pex15 binding alters the conformation of the Pex1/Pex6 N-terminal domains, we compared the negative-stain EM reconstructions of Pex1/Pex6 in the absence and presence of the Pex15 cytosolic portion. The Pex1/Pex6/Pex15 complex revealed additional density extending from the N-terminal domains of each Pex6 subunit (black arrowhead, Fig. 2b), as well as above the central pore of the hexamer (red arrow, Fig. 2b), but no Pex15-induced conformational changes of Pex1/Pex6 itself (Fig. 2b, Supplementary Figure 2). Guided by its curved shape and the orientation of its longest helix, the core-domain structure of Pex15 was docked into the difference density above the Pex6 N-terminal domains (Fig. 2c). Building upon the atomic model for Pex1/Pex6 proposed by Blok et al.^20^, this placement suggests that Pex15’s N-terminus binds between the N1 and N2 domains of Pex6 (Fig. 2c). We note that the 42 N-terminal residues of Pex15 that are not included in the crystal structure can be accommodated in the additional, unfilled density at the contact sites between Pex15 and Pex6. This orientation of Pex15 is also supported by previous in vivo studies alluding to the functional importance of Pex15’s N-terminal region^41,51^, as well as our observation that removal of Pex15’s N-terminal domain alters its apparent affinity for Pex1/Pex6 and competence for peroxisomal matrix protein import in vivo.

To verify that Pex15 contacts the Pex6 N1 and N2 domains, we performed HDX-MS to identify regions of Pex1/Pex6 that are shielded from the solvent by Pex15. Using an ATPase-deficient Pex1/Pex6 to eliminate any processing and assess only initial binding, we observed a peptide in the Pex6 N2 domain (aa 241–250, magenta peptide in Fig. 2c) and a 21,978 Da undigested fragment with increased protection from the solvent in the presence of Pex15 (Supplementary Figure 3, Supplementary Table 2). We hypothesized that this undigested fragment comprises aa 2–195 of His-Pex6 (green in Fig. 2c), because this region has a similar molecular weight (21,979 Da) and did not yield any individual peptides after peptic proteolysis (Supplementary Figure 3). In support of this hypothesis, pull-down binding assays showed that recombinant Pex6 N1 domain binds Pex15 (Supplementary Figure 4B). Additionally, mutations in the Pex6 N2 peptide (V245A and T246A) decreased the apparent affinity of Pex1/Pex6 for Pex15 1-309 from 0.6 to 2 μM as judged by the concentration-dependent inhibition of Pex1/Pex6 ATPase activity (Supplementary Figure 4A). Together, the negative stain EM, HDX-MS, ATPase, and pull-down-binding assays indicate that Pex15 binds Pex1/Pex6 at the interface between the Pex6 N1 and N2 domains. Notably, Pex15 can simultaneously occupy the N-terminal domains of all three Pex6 subunits in the hexamer, but it did not induce any large conformational changes in these N-terminal domains that could allosterically regulate Pex1/Pex6’s ATP hydrolysis. Instead, the additional density above the central pore of the complex (red arrow, Fig. 2b) suggests a potential engagement and processing of Pex15, which would alter ATPase activity through pore-loop contacts.

### Pex15 processing by Pex1/Pex6

**Fig. 3:**
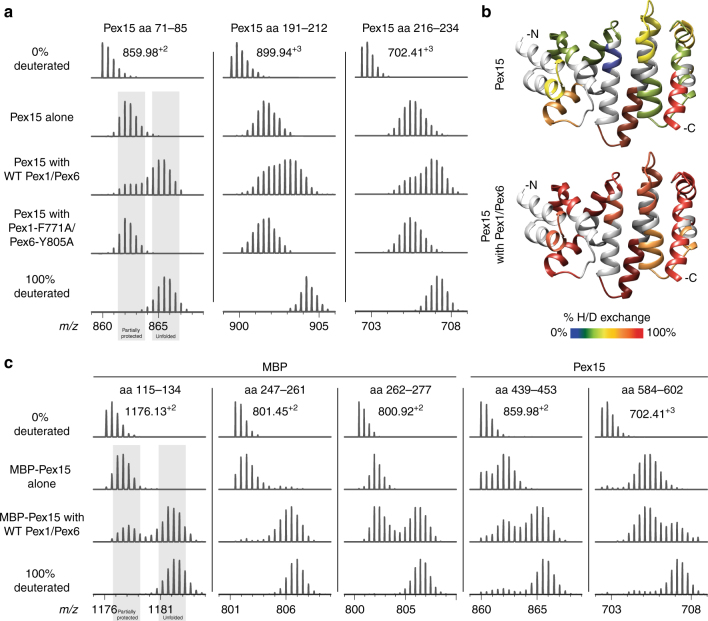
Pex1/Pex6 unfolding of Pex15 measured by HDX-MS. **a** Mass spectra of selected Pex15 peptides after a 60 s pre-incubation alone, with wild-type Pex1/Pex6, or with pore-loop mutant Pex1/Pex6 followed by 15 s of deuteration. Control spectra of no deuteration and 100% deuteration are shown for comparison. **b** The crystal structure of the Pex15 core domain is colored according to the relative deuteration levels of Pex15 peptides after a 30 s deuteration in isolation (top) or in the presence of wild-type Pex1/Pex6 motor (bottom). Regions without peptide coverage are colored light gray. **c** Mass spectra of selected peptides from an MBP-Pex15 fusion demonstrate that Pex1/Pex6 can unfold both MBP and Pex15 moieties. For each peptide, spectra are shown for MBP-Pex15 after 60 s incubation alone or in the presence of Pex1/Pex6, followed by a 15 s deuteration

To determine whether Pex15 is unfolded by Pex1/Pex6, we monitored its solvent accessibility by HDX-MS. While HDX-MS is commonly used to study protein unfolding^52^, it has recently been demonstrated to be a useful technique to monitor motor-mediated substrate unfolding^32,53^. Backbone amide hydrogens in the core region of Pex15 were protected from solvent when incubated in isolation or in the presence of pore-loop mutant Pex1/Pex6 (Fig. 3a) and pulse deuterated for 15 s. In contrast, after a 60 s incubation of Pex15 with wild-type Pex1/Pex6 in the presence of ATP, pulse-deuterated peptides from the core domain exhibited both a protected, partially deuterated state and an intermediate, more deuterated state (Fig. 3a). This behavior is a characteristic feature of global protein unfolding, which exposes multiple backbone amide hydrogens in a single event, and it suggests that Pex1/Pex6 mechanically unfolds the cytosolic domain of Pex15. By mapping the deuteration level of a series of peptides on the crystal structure of Pex15, it is apparent that Pex1/Pex6 can globally unfold the cytosolic domain of Pex15, exposing peptides throughout the core domain to the solvent (Fig. 3b, Supplementary Figure 5).

The crystal structure of Pex15 shows a single domain that we predicted would unfold cooperatively in one step. Consistently, measurement of the denaturant-induced equilibrium unfolding by circular dichroism exhibited a single transition with no indication of stable intermediates (Supplementary Figure 6A, 6B). Pex1/Pex6 could therefore accomplish the cooperative unfolding of Pex15 by tugging and releasing or by processively threading Pex15 through the central pore. To distinguish these mechanisms, we fused a maltose-binding protein (MBP) to the N-terminus of Pex15 to determine whether Pex1/Pex6 would unfold only Pex15 or also subsequently unfold MBP. The N-terminal MBP moiety had no effect on Pex15’s function in vivo or its inhibition of Pex1/Pex6’s ATPase activity in vitro (Supplementary Figure 6C, 6D). Using HDX-MS, we found that peptides from both Pex15 and MBP in an MBP–Pex15 fusion protein exhibited a new unfolded, more deuterated population when incubated with Pex1/Pex6, but not with buffer (Fig. 3c). This result confirms that substrate processing occurs by threading through the central pore, rather than by tug and release or conformational changes of Pex1/Pex6’s N-terminal domains.

**Fig. 4:**
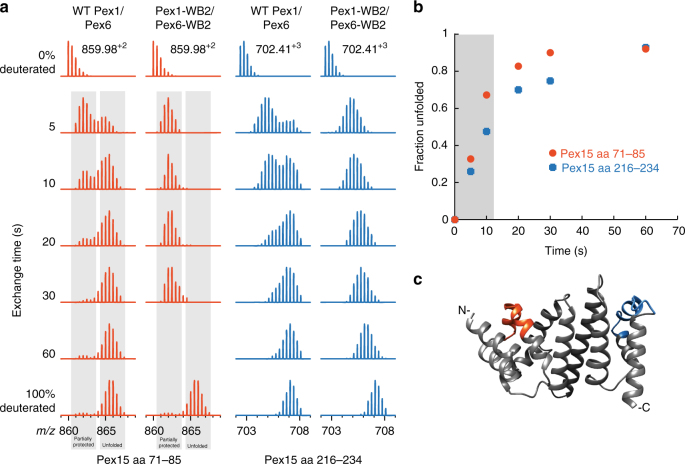
Pex1/Pex6 unfolding of Pex15 reveals EX1 kinetics. **a** Mass spectra of two Pex15 peptides after variable times of simultaneous deuteration and incubation with wild-type and WB2-mutant Pex1/Pex6. Control spectra of no deuteration and 100% deuteration are shown for comparison. The spectrum for Pex15 aa 71–85 after 60 s with WB2-mutant Pex1/Pex6 was not observed. **b** The fraction of Pex15 unfolded by Pex1/Pex6 over time allows for an estimation of the initial linear rate of Pex15 unfolding (gray box). **c** Peptides used for analysis in panels **a** and **b** mapped onto the crystal structure of the Pex15 core domain

To determine the rate of Pex15 unfolding by Pex1/Pex6, we altered our experimental setup such that Pex15 was simultaneously exposed to both the motor and the deuterated solvent, rather than pre-incubated with the motor and subsequently deuterated. Under these conditions, Pex15 was continuously deuterated as it unfolded, and we were able to calculate the initial rate of unfolding to be ~60 min^−1^ for peptide 1 (aa 71–85) and ~42 min^−1^ for peptide 2 (aa 216–234) (Fig. 4). At this rate, all available Pex15 in our sample is unfolded by Pex1/Pex6 within 60 s. Considering that our previous experiments (Fig. 3a), which used a 15-s deuteration after a 60-s pre-incubation with Pex1/Pex6, showed a considerable population of protected Pex15, we surmise that Pex15 can rapidly refold after Pex1/Pex6 processing.

Under identical conditions as in the HDX-MS experiment, recombinant Pex1/Pex6 in the presence of Pex15 hydrolyzed ~3000 ATP per minute (Supplementary Figure 6E). Considering the unfolding rate of ~60 Pex15 per minute, Pex1/Pex6 thus utilizes ~50 ATP to process one Pex15 molecule. Since some of this ATP is likely consumed during mechanical unfolding, we conclude that the motor translocates the 309-residue polypeptide of our cytosolic Pex15 construct with a step size of at least seven amino acids per ATP. This step size is consistent with single-molecule measurements of the AAA proteases ClpXP and ClpAP, which exhibited maximal translocation increments of 10 and 8 amino acids per ATP, respectively^54,55^.

### In vitro and in vivo requirements for Pex1/Pex6 activity

**Fig. 5:**
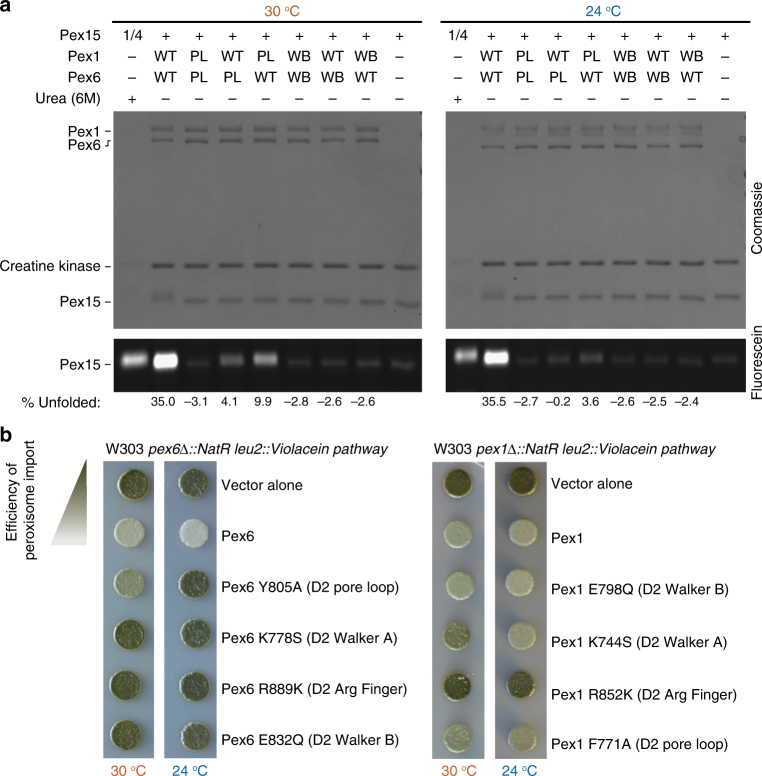
Effects of Pex1 and Pex6 mutations on Pex15 unfolding in vitro and peroxisomal matrix-protein import in vivo. **a** Unfolding of the Pex15 cytosolic domain (aa 1–309) by wild-type and mutant Pex1/Pex6 was assessed by SDS-PAGE after fluorescein-5-maleimide labeling of Pex15’s buried cysteines. The percent of unfolded Pex15 (mean of *n* = 3) was determined by normalization to the basal labeling of Pex15 alone or in the presence of 6 M urea. For the urea control, a quarter of the total reaction was loaded on the gel. **b** The efficiency of peroxisome matrix-protein import in the presence of mutant Pex1 and Pex6 was tested by comparing the colony color of strains containing the violacein pathway with VioE targeted to the peroxisome. Cells with efficient protein import remain white, while defects in peroxisomal import lead to a green color development. Colonies shown are representative of *n* = 3 biological replicates

Having established that Pex1/Pex6 unfolds Pex15 in vitro by threading through the central pore, we wanted to determine whether the motor requirements in vitro reflect the same requirements for peroxisome matrix-protein import in vivo. In order to measure the unfolding of Pex15 by Pex1/Pex6 with higher throughput, we took advantage of the observation that all of the cysteine residues of Pex15 reside within the folded core and are inaccessible to solvent. The percent of unfolded Pex15 after incubation with Pex1/Pex6 can therefore be quantified by pulse-labeling of exposed cysteines with fluorescein-5-maleimide (F5M) (Fig. 5a). To compare the requirements for in vitro processing of Pex15 with the efficiency of peroxisomal matrix protein import in vivo, we used a colorimetric assay that is capable of detecting subtle changes in the efficiency of peroxisome import in yeast (Fig. 5b). It employs the violacein pathway from *Chromobacterium violaceum*, which converts tryptophan into the green pigment prodeoxyviolacein. In this assay, the final enzyme of the pathway, VioE, is tagged with a PTS1 signal (SKL), and is therefore rapidly imported into peroxisomes and separated from its colorless cytosolic substrate^56^. Yeast cells with intact peroxisomes and efficient matrix-protein import consequently are white, while yeast cells with impaired import accumulate cytosolic VioE-SKL and turn green due to formation of prodeoxyviolacein.

For the in vitro unfolding assay, we optimized the maleimide-labeling time to maximize the difference between the labeling of folded Pex15 in isolation and unfolded Pex15 in the presence of 6 M urea. Consistent with our HDX-MS experiments, we observed strong fluorescein labeling and thus robust unfolding of Pex15 in the presence of wild-type Pex1/Pex6, which we then compared with Pex1/Pex6 mutants (Fig. 5a). Interestingly, Pex15 unfolding by the D2 pore-loop mutants was temperature dependent: mutations in the D2 pore loop of either Pex1 or Pex6 reduced Pex15 unfolding at 30 °C, and further reduced it at 24 °C. Pex1/Pex6 with D2 pore-loop mutations in both subunits was unable to unfold Pex15, which is consistent with our results by HDX-MS (Fig. 3a). While the importance of the D2 pore loops had previously been observed in vivo, a temperature-dependent phenotype as predicted from our in vitro assay had not yet been reported. Using the in vivo colorimetric assay for peroxisomal matrix-protein import, we detected a partial defect for the Pex6 D2 pore-loop mutant at 30 °C, but complete deficiency at 24 °C (Fig. 5b). The consistency of this cold-sensitivity of the Pex6 pore-loop mutant therefore suggests that Pex1/Pex6 uses the same unfolding mechanism in vitro and in vivo. We note that Cdc48/p97 was also originally identified as a cold-sensitive mutant^57^, which may suggest a particular sensitivity of unfoldases to changes in the stability of their protein substrates or the reduction of motor speed and thus pulling frequency at lower temperature.

Since pore-loop movement is driven by ATP hydrolysis, we next assessed the activities of ATPase-deficient Pex1/Pex6 mutants. As expected, in vitro unfolding and maleimide labeling of Pex15 depended on ATPase activity of the motor, and it did not occur when Pex1, Pex6, or both contained Walker-B mutations in the D2 domains. The in vivo colorimetric assay confirmed the importance of Pex6 ATP hydrolysis for efficient peroxisomal import: the Pex6 D2 WA and WB motifs, and the Pex1 D2 arginine finger are essential for import of VioE-SKL. However, the Pex1 D2 Walker B (WB2) mutant imported VioE-SKL similar to wild-type Pex1/Pex6, even though it failed to unfold Pex15 in vitro, as determined by maleimide labeling, as well as HDX-MS (Supplementary Figure 7). There are several plausible explanations for this inconsistency. For instance, our in vitro assays may not be sensitive enough to detect slow rates of Pex15 unfolding that could be sufficient in vivo. Another possibility is that either Pex15 is more readily unfoldable in the context of the cell or Pex1/Pex6 has other, more labile substrates with critical roles in matrix-protein import.

### In vitro and in vivo requirements for Pex15

**Fig. 6:**
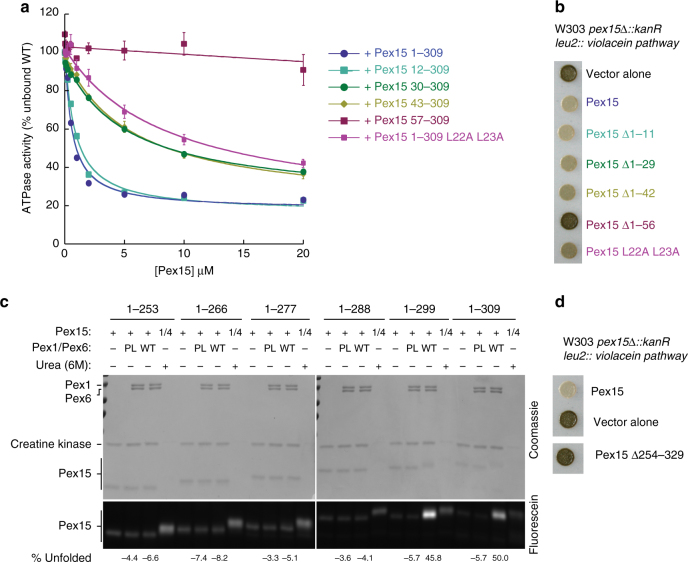
The efficiency of peroxisomal matrix-protein import correlates with the apparent Pex15-binding affinity for Pex1/Pex6. **a** Relative affinities of Pex15 N-terminal truncations for Pex1/Pex6 were determined by measuring the apparent *K*_D_’s for inhibition of Pex1/Pex6 ATPase activity (fit parameters in Supplementary Table 3). ATPase data shown are the mean±s.d. for *n* = 3 technical replicates. **b** and **d** The efficiency of peroxisomal matrix-protein import was assessed using the colorimetric assay described in Fig. 5b. Colonies shown are representative of *n* = 3 biological replicates. **c** Fluorescein-maleimide labeling of internal cysteines of Pex15 C-terminal truncation mutants in isolation and in the presence of wild-type and pore-loop mutant Pex1/Pex6. The % unfolded represent the mean of *n* = 3 technical replicates, normalized to the levels of basal labeling or labeling in the presence of 6 M urea

To test if the features of Pex15 that are important for Pex1/Pex6 binding and engagement in vitro are also critical in vivo for peroxisomal matrix-protein import, we compared the unfolding of Pex15 mutants in our maleimide-labeling assay with their ability to support efficient protein import in vivo. As indicated by our structural studies, the Pex15 N-terminal region is important for binding Pex1/Pex6 (Fig. 2). Indeed, we found that truncations from Pex15’s N-terminus decreased the apparent *K*_D_ in ATPase inhibition (Fig. 6a, Supplementary Table 3), with a step-wise reduction in affinity that directly correlated with the impairment of peroxisomal matrix-protein import in vivo (Fig. 6b). Removal of the first 12 amino acids from the N-terminus of Pex15 did not change Pex1/Pex6 binding and consequently showed no in vivo effect. However, deletion of residues 1–29 or 1–42 reduced the apparent affinity (Fig. 6a) and led to partial defects in VioE-SKL import (Fig. 6b) suggesting that aa 12–29 contain part of the Pex6-binding motif. Previous mutational analysis indicated that Pex15 L22 was important in vivo^41^, and indeed we found that mutation of L22 and L23 to alanine led to a reduction in apparent affinity for Pex1/Pex6 equivalent to the 1–29 or 1–42 truncations. The Δ1–56 mutant, which lacks the first helix in the crystallized core domain of Pex15, did not inhibit Pex1/Pex6 ATPase activity in vitro or support matrix-protein import in vivo (Fig. 6b). These data thus confirm that Pex15 uses its N-terminal flexible region to bind between the N-terminal domains of Pex6, as indicated by our EM reconstruction for the Pex1/Pex6/Pex15 complex (Fig. 2), and that this interaction is essential for efficient peroxisomal matrix protein import.

Consistent with the N-terminus of Pex15 mediating binding to Pex1/Pex6, we found that truncations from the C-terminus did not impair Pex15 binding: both MBP-Pex15 1-309 and MBP-Pex15 1-253 interacted with Pex1/Pex6 in pull-down binding assays (Supplementary Figure 8A). However, only the variant with the longer C-terminal tail, MBP-Pex15 1-309, was engaged and unfolded (Supplementary Figure 8B). A series of C-terminal truncation mutants revealed a sharp disparity between Pex15 1-299, which is rapidly unfolded and strongly inhibits Pex1/Pex6 ATPase activity, and Pex15 1-288, which is not processed by the motor (Fig. 6c, Supplementary Figure 8B). These data suggest a strict dependence of mechanical processing on a long C-terminal tail on Pex15, which is likely required to bridge the distance between the Pex15-binding site on the N-terminal domains of Pex6 and the D2 pore-loop residues for engagement and threading.

To test this dependence on length, we attempted to recover Pex1/Pex6 engagement of the Pex15 1-288 truncation mutant by extending its C-terminus through the addition or insertion of a glycine-serine segment. Even though these extensions did not restore the inhibition of Pex1/Pex6’s ATPase activity (Supplementary Figure 8D), they partially recovered Pex1/Pex6-mediated unfolding of Pex15 (Supplementary Figure 8C), indicating that the length of the C-terminal segment is an important requirement for motor engagement. In addition, the sequence of the tail may determine the motor “grip” on a substrate. The glycine-serine segment is expected to lower the unfolding efficiency of Pex1/Pex6, but may also reduce motor stalling in front of the mechanical barrier and thus alleviate ATPase inhibition^58–60^.

In vivo Pex15 is tail-anchored, with the long disordered region preceding its transmembrane domain. Pex1/Pex6 would therefore have to engage this disordered region as a loop rather than as a free C-terminus. Consistent with this model, we found that a Pex15 variant without the C-terminal disordered region did not support the import of VioE-SKL (Fig. 6d).

### Pex15 links Pex1/Pex6 with Pex5/Pex14

**Fig. 7:**
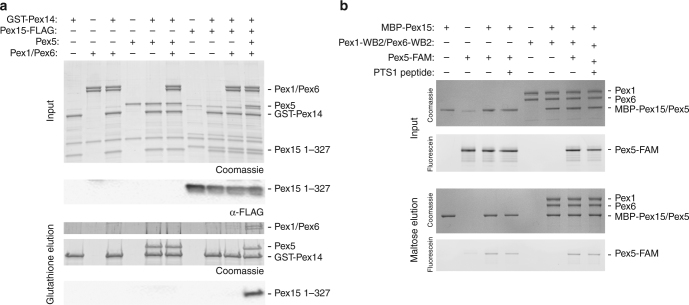
Pex15 bridges Pex5/Pex14 with Pex1/Pex6. **a** A pull-down binding assay of GST-Pex14 with Pex5, Pex15 1-327-FLAG, and Pex1/Pex6 in the presence of ATP shows that Pex1/Pex6 associates indirectly with Pex14 through an interaction requiring Pex5 and Pex15. Pex15-FLAG was detected by an anti-FLAG western blot on the input and elution. **b** A pull-down binding assay of MBP-Pex15 1-327 with Pex1/Pex6-WB2 and fluorescein labeled Pex5 with and without a PTS1 peptide. MBP-Pex15 and Pex5 migrate similarly, so the presence of Pex5 was determined by imaging its fluorescein label. MBP alone does not bind Pex5 (Supplementary Figure 9)

While Pex15 binding to Pex1/Pex6 is clearly important for peroxisomal matrix-protein import in vivo, it is possible that Pex1/Pex6 processes other substrates, and Pex15 plays a primary role in mediating their interaction or co-localization. For instance, Pex1/Pex6 has been proposed to extract the Pex5 cargo receptor from the peroxisomal membrane to recycle it for subsequent rounds of matrix-protein import, but there has been little evidence of direct interaction between Pex1/Pex6 and Pex5. We observed no pore-loop dependent effect of Pex5 on Pex1/Pex6’s ATPase activity, even with a linear ubiquitin fusion, and pull-down binding assays of Pex5 and Pex14 revealed no direct interactions with Pex1/Pex6 (Fig. 7a, see also Supplementary Figure 11). Tamura et al. observed that the human Pex15 homolog, Pex26, could bind both Pex5 and Pex14^51^, and we hypothesized that Pex15 might mediate a similar interaction between Pex1/Pex6 and Pex5. Indeed, we found that Pex1/Pex6 pulled-down with Pex5 and Pex14 only in the presence of Pex15 (Fig. 7a). Furthermore, we observed direct binding between MBP-Pex15 and fluorescein-labeled Pex5, but not MBP-Pex6/Pex1 (Supplementary Figure 9, Fig. 7b). The interaction between MBP-Pex15 and Pex5 was not altered by the presence of Pex1/Pex6 or by a PTS1 peptide (Fig. 7b). Interestingly, similar to the unstructured regions of Pex15 that are critical for binding and processing by Pex1/Pex6, Pex5 also has a long, flexible N-terminal domain^61^ (Supplementary Figure 10). We were unable, however, to detect any unfolding of Pex5 in the presence of Pex1/Pex6 and Pex15 by HDX-MS (Supplementary Figure 10), so the context requirements for potential Pex5 engagement and processing by Pex1/Pex6 remain unclear.

## Discussion

Pex1 and Pex6 form a heterohexameric motor protein related to NSF, which disassembles post-fusion SNARE complexes, and Cdc48/p97, which can unfold proteins, extract them from membranes, and disassemble protein complexes. These Type 2 AAA-ATPases that contain two ATPase rings, D1 and D2, and substrate interacting N-terminal domains, typically convert the energy of ATP hydrolysis into mechanical force by either driving conformational changes in their N-terminal domains or movement of hydrophobic central pore loops. The large conformational changes in the N-terminal domains, such as those used by NSF, appear to depend on only a single round of ATP hydrolysis to pull apart protein complexes^62^. In contrast, substrate processing by movements of the hydrophobic central pore loops, such as those used by the Clp unfoldases, requires successive rounds of ATP hydrolysis to processively thread a protein through the central pore, thereby leading to protein unfolding, extraction from membranes, and disassembly of complexes. Cdc48/p97 performs a combination of these mechanisms, both mechanically threading substrates through the central pore and exhibiting N-terminal domain movements that may mediate co-factor engagement or activity^31^.

Pex1 and Pex6 are essential for proper peroxisome biogenesis and maintenance and have been proposed to function similarly to NSF in pre-peroxisomal vesicle fusion and/or similarly to Cdc48/p97 in the extraction of ubiquitinated proteins from membranes. In the latter, better-established model, Pex1/Pex6 extract the ubiquitinated cargo receptor Pex5 from the peroxisomal membrane to recycle it for continuous rounds of peroxisomal matrix protein import. While Pex1/Pex6 ATPase activity has been correlated with Pex5 release from purified membranes in vitro^17^, no direct processing or robust interaction with Pex5 has been observed. Furthermore, while no nucleotide-dependent conformational changes of the Pex1 or Pex6 N-terminal domains have been detected, it remained unclear whether Pex1/Pex6 could use its central pore loops to unfold substrates by mechanical threading.

Here, we showed that the Pex1/Pex6 hexamer is an unfoldase that processively threads its substrates through the central pore, which is consistent with a role in extracting proteins from the peroxisomal membrane. However, we did not observe this activity through any direct processing of Pex1/Pex6’s expected substrate, Pex5, but rather uncovered the unfolding of the cytoplasmic domain of Pex15, Pex1/Pex6’s receptor on the peroxisomal membrane. This raises questions about the identity of Pex1/Pex6’s native substrates in vivo and suggests a model in which Pex5 extraction or release from the membrane could be an indirect consequence of Pex1/Pex6-mediated unfolding of a different Pex protein, such as Pex15.

Our data suggest that Pex1/Pex6 uses its N-terminal domains to initially bind its substrates, which are subsequently engaged by the D2 pore loops and translocated through ATP-hydrolysis-driven  movements of the pore loops. Substrate proteins thus require both affinity for Pex1/Pex6 and a disordered region long enough to reach from the binding sites on the N-terminal domains through the inactive D1 ring to the pore loops in the active D2 ring. Other Pex proteins, including Pex5, Pex13, and Pex14, contain intrinsically disordered regions that could be engaged by Pex1/Pex6. While we did not observe Pex1/Pex6 processing of Pex5 or any influence of Pex5 or a linear ubiquitin-Pex5 fusion on Pex1/Pex6 ATPase activity, it is possible that Pex5 recognition requires additional modifications, such as site-specific mono-ubiquitination^14,15^, or binding partners that we have thus far been unable to recapitulate in vitro. We note that the Pex1 and Pex6 subunits have unique N-terminal domains that could bind different sets of substrates, perhaps allowing Pex1/Pex6 to process a range of proteins and therefore play multiple roles in peroxisome function. We expect all these roles, however, to rely on the hydrolysis-driven conformational changes and substrate translocation by the central pore loops.

Pex15, whose cytoplasmic domain we found to be an in vitro substrate of Pex1/Pex6, binds the Pex6 N-terminal domains and presents a long, disordered C-terminal tail for engagement and processing in vitro. This observation readily agrees with earlier reported data, but invites a new interpretation. We previously observed that Pex15 binding inhibits Pex1/Pex6’s ATPase activity^18^, which we proposed could act through an interaction between Pex6’s N-terminal domain and the Pex1 D2 ATPase. Instead, we now found that Pex15 inhibition of Pex1/Pex6 ATPase activity is dependent on the central pore loops and correlates with Pex15’s engagement as a substrate in the central pore and not as an external binding partner. Other labs reported that Pex1/Pex6 ATPase activity reduces Pex15 binding and the motor’s association with the peroxisomal membrane^42^. These observations had led to the model that Pex15 binds in a nucleotide-dependent manner to the Pex6 N-terminal domains, which allows Pex1/Pex6 to cycle on and off the peroxisomal membrane controlled through its ATPase activity. However, given our observation that Pex1/Pex6 unfolds Pex15, we proffer the alternative model that Pex15’s initial binding to the Pex6 N-terminal domains is independent of the nucleotide state and that Pex1/Pex6 ATPase activity reduces the motor’s affinity for Pex15 because it actively translocates, unfolds, and dissociates Pex15. The observation that the Pex6 Walker B mutant increases the association of Pex1/Pex6 with the peroxisomal membrane and also a number of Pex proteins including Pex15 and Pex5^42^ suggests that the ATPase activity of Pex1/Pex6 is required to unfold one or more of these Pex proteins and permit complex dissociation. It remains unclear, however, which of Pex15 or other Pex proteins are Pex1/Pex6 substrates in vivo.

In our in vitro assay, the cytoplasmic domain of Pex15 is engaged through its disordered C-terminal region, which would be followed by a transmembrane helix in vivo. The Pex1/Pex6 motor would thus have to engage this region as a loop and eventually release Pex15 by extraction of its transmembrane domain or by disassembly of the ATPase hexamer. In vivo, we found that peroxisomal matrix-protein import depends on Pex15’s disordered C-terminal region, supporting the model that Pex15 is an in vivo substrate. Surprisingly, a Pex1-WB2/Pex6 mutant that could not unfold Pex15 in vitro had no defect in our colorimetric assay for peroxisomal matrix-protein import in vivo. Others have observed that Pex1-WB2 mutants can grow on oleic acid^19^, and in human cells the Pex1-WB2 mutant had no defect for the peroxisomal import of GFP-SKL, albeit showing less efficient import of catalase^63^. However, consistent with our observations that Pex1-WB2/Pex6 has lower ATPase activity^18^ and fails to unfold Pex15 in vitro, others found that the Pex1-WB2 mutant is also impaired for Pex5 extraction from membranes^17^ and dissociating Pex26 from Pex14 in human cells^51^. This inconsistency between in vitro and in vivo experiments remains to be resolved, but could possibly be caused by a difference in folding environments that makes substrates easier to unfold in vivo.

We and others have observed that efficient peroxisomal matrix-protein import in vivo depends on Pex15’s affinity for Pex1/Pex6. Thus, even if Pex15 is not a substrate itself, it may serve as an important co-factor for recruiting other substrates to Pex1/Pex6. We found that Pex15 can directly bind Pex5 and link the Pex5/Pex14 complex to Pex1/Pex6. Pex15 may thus deliver substrates, rather than just recruiting Pex1/Pex6 to the peroxisomal membrane and the nearby proteins. Since Pex15 has been shown to be mistargeted to the mitochondrial membrane^64,65^, specific substrate selection by Pex1/Pex6 seems critical to prevent aberrant unfolding of alternative substrates based solely on proximity.

**Fig. 8:**
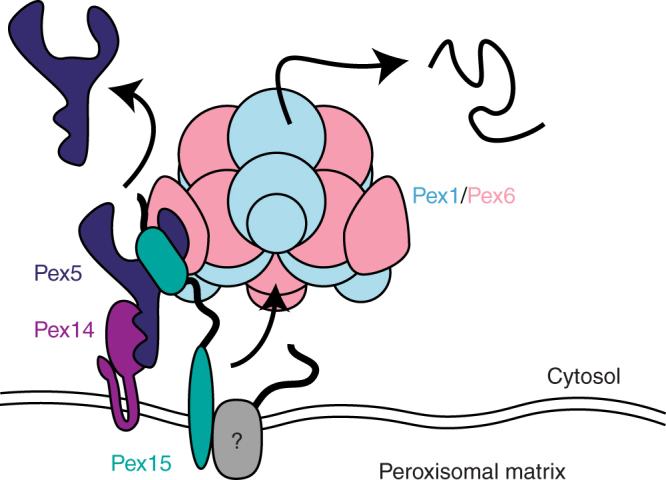
Model for Pex1/Pex6 activity at the peroxisomal membrane. Pex1/Pex6 is recruited to the peroxisomal membrane by binding to Pex15, which mediates its interaction with Pex5, Pex14, and potentially other Pex proteins (gray). Pex1/Pex6 ATPase activity at the peroxisome membrane leads to unfolding of one or more members of this complex, which either directly or indirectly causes Pex5 extraction from the peroxisomal membrane

In summary, our data establish that Pex1/Pex6 is a protein unfoldase that uses aromatic pore-loop residues to processively thread its substrates through the central pore. Based on these findings, the current model (Fig. 8) is that Pex15 recruits Pex1/Pex6 to the peroxisomal membrane and directly links it to Pex5/Pex14 and potentially other Pex proteins. Pex1/Pex6 may then engage and unfold one or more Pex proteins that contain long, disordered regions, leading either directly or indirectly to Pex5 extraction from the membrane, resetting of the peroxisomal import machinery, and disassembly of the Pex1/Pex6 substrate complex. Our work sets the stage for future investigations into the identity of endogenous Pex1/Pex6 substrates and the mechanisms that couple protein unfolding on the cytosolic surface of peroxisomes with the import of peroxisomal matrix proteins.

## Methods

### Cloning

Coding regions for Pex proteins were amplified from *S. cerevisiae* W303 and cloned into bacterial and yeast plasmids using primers listed in Supplementary Table 4.

### Pex1/Pex6 purification

Pex1-FLAG and His-Pex6 wild type and mutant complexes were co-expressed in *E. coli* BL21* (ThermoFisher Scientific) from the pETDuet and pCOLADuet vectors. The expression strain was grown in 6 L of DYT (16 g tryptone, 10 g yeast extract, 5 g NaCl) and appropriate antibiotics at 30 °C and induced at OD_600_ = 0.6–0.9 with IPTG (final concentration *C*_f_=0.3 mM) before overnight incubation at 18 °C. The *E. coli* were harvested at 6000×*g* for 20 min at 4 °C, and the pellet was resuspended in Ni–A buffer (25 mM HEPES pH 7.6, 100 mM NaCl, 100 mM KCl, 10% glycerol, 10 mM MgCl_2_, 0.5 mM EDTA, 20 mM imidazole) with benzonase, lysozyme (0.2 mg mL^−1^), and protease inhibitors and frozen at −80 °C. Cells were lysed by sonication at 90 mA with 15 s pulses on, 90 s off for a total of 120 s on. Cell debris and unlysed cells were pelleted at 30,000×*g*, and the supernatant was transferred to conical tubes containing 5 mL of pre-washed Ni-NTA resin (HisPur Ni-NTA Resin, Thermo). The cell lysate and agarose were incubated with gentle rocking for 1–2 h at 4 °C before the agarose was batch washed with 2 × 50 mL washes of Ni_A with 1 mM ATP. After the batch washes, the agarose was poured into a gravity flow column and washed until the flowthrough contained no protein, as judged by a Bradford assay. The bound protein was then eluted with Ni_B buffer (25 mM HEPES pH 7.6, 100 mM NaCl, 100 mM KCl, 10% glycerol, 500 mM imidazole) with 1 mM ATP, and the elution was added to re-suspended and pre-washed anti-FLAG M2 affinity resin (Sigma) for 2 h of batch binding at 4 °C. After 2 h, the anti-FLAG affinity resin was poured into a gravity flow column, and washed with 50 mL of Ni_A with 1 mM ATP. The bound protein was eluted with Ni_A with 1 mM ATP and 0.3 mg mL^−1^ FLAG peptide and concentrated on a 100 MWCO spin concentrator before snap-freezing in liquid nitrogen. To separate the Pex1-FLAG/His-Pex6 hexamer from other oligomers, the concentrated FLAG elution was loaded on a Superose6 size exclusion column equilibrated in GF buffer (60 mM HEPES pH 7.6, 50 mM NaCl, 50 mM KCl, 10% glycerol, 10 mM MgCl_2_, 0.5 mM EDTA) with 1 mM ATP. The concentration of protein was determined by a Bradford assay. The amino acid changes in the ATPase motifs of Pex1 and Pex6 are as follows: Pex1-Walker B D2 (E798Q), Pex6-WB D2 (E832Q), Pex1-D2 poreloop (F771A), and Pex6-D2 pore loop (Y805A).

For Pex1-Strep/His-Pex6, we followed the same protocol through the elution from the Ni-NTA agarose, at which point the elution was batch bound to Streptactin Superflow Plus resin (QIAGEN) and eluted with Ni_A buffer with 1 mM ATP and 2.5 mM desthiobiotin. Pex1-Strep/His-Pex6 was used for the HDX-MS experiments and related ATPase activity assay and pull-down binding assays. Pex1-FLAG/His-Pex6 was used for all other experiments including negative stain EM, ATPase assays with Pex15 constructs, and fluorescein maleimide labeling experiments.

His-Pex1-FLAG and His-Pex6-FLAG were each purified individually by solo expression in BL21* *E. coli*. The cells were induced and harvested in the same way as described for hexameric Pex1/Pex6. Pex1 and Pex6 were then purified with a first-step Ni-NTA column and a second-step FLAG affinity column. His-Pex6 1-215 was expressed in BL21* *E. coli* in the same manner as the full-length protein and was purified in the same way through the first Ni-NTA affinity column.

### Purification of Ubp15

Ubp15 was expressed as a GST-Ubp15-His fusion protein from pGEX6P-2 in *E. coli* BL21*. Expression was induced at OD_600_ = 0.6 with IPTG (*C*_f_=0.3 mM) overnight at 18 °C. The harvested cells were resuspended in Ni_A buffer supplemented with protease inhibitors, 2 mg mL^−1^ lyzosyme, and benzonase, and lysed with 2 min of sonication on ice. The lysed cells were centrifuged for 30 min at 30,000×*g*, and the supernatant was batch bound to Ni-NTA resin for 30 min, rotating end-over-end. The protein-bound resin was washed with Ni_A buffer for ~20 CVs. Ubp15 was eluted with 10 CVs of Ni_B buffer, and the eluate was batch bound to glutathione agarose (Pierce). The slurry was poured into a gravity flow column and washed with 20 CVs of wash buffer (60 mM HEPES pH 8, 100 mM KCl, 100 mM NaCl, 10% glycerol), before the GST bound protein was eluted with 20 mM reduced glutathione in the same buffer. The GST tag was cleaved from Ubp15 in an overnight incubation with PreScission protease at 4 °C. Ubp15 cleaved from GST was collected as the flowthrough of a second glutathione agarose column, concentrated, and run over a Superose 6 increase sizing column into GF buffer.

### Pex15 protein purification

Protein was expressed in *E. coli* BL21* cells in DYT. Cultures were grown shaking at 37 °C and were induced at OD_600_ = 0.6 by the addition of IPTG (*C*_f_=0.3 mM), then incubated shaking for ~18 h at 18 °C. Cells were harvested by centrifugation at 6000×*g* for 20 min at 4 °C, then resuspended in Ni_A buffer supplemented with protease inhibitors: leupeptin, pepstatin, aprotinin, 2 mg mL^−1^ lysozyme, and benzonase (Novagen). Resuspended cell pellets were stored at −80 °C until thawed for purification. Thawed cells were sonicated on ice for 2 min, then the lysate was clarified by centrifugation for 30 min at 30,000×*g*. The subsequent soluble extract was batch bound to Ni-NTA resin for 30 min, rotating end-over-end. The protein-bound resin was washed with Ni_A buffer for ~40 CVs. Pex15 was eluted with 10 CVs of Ni_B buffer. The affinity purification tags were cleaved by PreScission protease overnight while dialyzing into Ni_A buffer. Uncleaved protein Pex15-His and PreScission Protease were removed by incubation with Ni-NTA resin. The resulting flow-through was concentrated and run on a HiLoad 16/60 Superdex 200  column (GE Life Sciences) into GF Buffer. MBP-Pex15-FLAG-His constructs were purified with His and FLAG affinity steps as for Pex1/Pex6, but without ATP, so that the maltose-binding protein could be used for pull-down binding assays.

### Selenomethionine-Pex15 expression for crystallography

Cultures were grown shaking in minimal media at 37 °C. At OD_600_ = 0.5, dry stocks of amino acids were added, including selenomethionine (SeMet; 75 mg/L). After addition of amino acids, cultures were grown for an additional 15 min before induction with IPTG (*C*_f_ = 0.3 mM) and overnight growth at 18 °C. SeMet-containing Pex15 protein was prepped as described above. During size exclusion chromatography, SeMet-Pex15 was buffer exchanged into a minimal buffer (20 mM HEPES pH 7.6, 50 mM NaCl, 50 mM KCl, 0.5 mM TCEP).

### Pex5 purification

FLAG-Pex5-His and His-Ub-Pex5-FLAG were expressed in *E. coli* BL21* cells in DYT. Cultures were grown shaking at 30 °C and were induced at ~OD_600_ = 0.6 by the addition of IPTG (*C*_f_=0.3 mM), then incubated shaking for ~5 h at 30 °C. Cells were lysed by sonication and the 25,000×*g* supernatant was batch bound to Ni-NTA resin for 30 min. The protein-bound resin was washed with Ni_A buffer for ~40 CVs. Pex5 was eluted with 10 CVs of Ni_B buffer. Pex5 was then repeatedly flowed over anti-FLAG affinity resin 5–7 times, before being eluted with ~10 CVs of Ni_A buffer containing 0.15 mg mL^−1^ FLAG peptide. The Pex5 eluted from FLAG resin was concentrated and run on a Superdex 200 increase 10/300 GL size exclusion column (GE Life Sciences).

### Pex5 conjugation to FAM

For Pex5 constructs containing a Sortase cleavage site (LPETGG) just upstream of the C-terminal 6xHis tag, Pex5 (30 μM) were incubated with sortase (5 μM) and fluorescein-conjugated peptide GGGK-FAM peptide (50 μM, Elim BioPharm) for 2 h at 4 °C. The mixture was run back over fresh Ni-NTA resin to remove uncleaved protein, and the resulting flow-through was concentrated and run on a Superdex 200 increase 10/300 GL size exclusion column (GE Life Sciences).

### Pex14 purification

Pex14 was expressed as an N-terminal GST fusion protein. GST-Pex14 was expressed in *E. coli* BL21* cells in 6 L of DYT. Cultures were grown shaking at 37 °C and were induced at OD_600_ = 0.6 by the addition of IPTG (*C*_f_=0.3 mM), then incubated shaking for 5 h at 30 °C. Cells were harvested by centrifugation at 6000×*g* for 20 min at 4 °C, resuspended in Ni_A buffer supplemented with 0.1% TritonX-100, lysozyme, benzonase, and protease inhibitors, and stored frozen at −80 °C. Thawed cells were lysed by sonication on ice for 2 min, and then lysate was clarified by centrifugation for 30 min at 25,000×*g*. The subsequent soluble extract was batch bound to glutathione agarose (Pierce) for 30 min, rotating end-over-end. The protein-bound resin was washed with Ni_A buffer with 0.1% TritonX-100 for ~40 CVs. Pex14 was eluted with 10 CVs of Ni_A buffer with 0.1% TritonX-100 and 20 mM reduced glutathione. The elution was concentrated and run on a Superose6 Increase 10/300 GL size exclusion column (GE Life Sciences).

### Circular dichroism

Circular dichroism was measured using an Aviv CD spectrophotometer, model 140. Pex15 protein was dialyzed into CD buffer (50 mM PBS pH 7.6, 50 mM KCl) overnight using a 10,000 MWCO Slide-A-Lyzer MINI Dialysis Unit (Thermo) before being diluted to a final concentration of 0.04 mg mL^−1^ and loaded into an Agilent Technologies Open-top UV quartz cell (10 mm pathlength, 3.0 mL volume). CD buffer without any Pex15 was used for subtraction of the baseline signal in later calculations. CD spectra were measured between 300 and 200 nm with 1 nm intervals and a 1 nm bandwidth. Each measurement was an average of the signal collected for 5 s at each wavelength. The mean residue ellipticity (MRE) was calculated by subtracting the buffer-alone CD signal from the Pex15 CD signal (*θ*_Pex15_−*θ*_buffer_).

CD measurements of the chemically induced equilibrium unfolding of Pex15 1-309 was performed at 30 °C. Samples with increasing concentrations of urea in CD buffer were allowed to equilibrate overnight. Before recording the CD signal at 222 nm for 60 s each sample was temperature-equilibrated in the instrument’s cuvette holder at 30 °C for 60 s while mixing. The average value for the 60 s data collection was plotted against the concentration of urea, which was determined for each sample by measuring its refractive index.

### Limited proteolysis

In 100 mM Tris (pH 8.0) buffer, 0.75 μg/μL Pex15 protein was digested with 0.002 or 0.02 μg/μL trypsin protease (Sigma) for 10 min at 23 °C. Immediately prior to quenching, 10% of the reaction volume was removed and quenched with the addition of PMSF (*C*_f_=0.7 mM) and SDS-PAGE sample buffer (12.5 mM Tris pH 6.8, 10% glycerol, 2% SDS, 0.005% bromophenol blue). This sample was run on an SDS-PAGE gel for analysis. The remaining reaction mixture was quenched with an equal volume of 6 M guanidinium–HCl and flash frozen until analyzed by mass spectrometry. Mass spectrometry revealed four prominent cleavage sites after residues K36, R42, K253, and K255.

### Crystal-structure determination of Pex15

Crystal conditions were screened using JCSG screens I-IV (Qiagen). Initial screens were set at 18 °C in sitting-well Intelli-Plate 96-3 LVR (Hampton Research). Two hundred microliters of 5, 10, and 15 mg mL^−1^ Pex15 were mixed with 200 μL of screen solution using a mosquito liquid-handling robot (TTP Labtech). Promising conditions were further screened and scaled up 10-fold to 4 μL hanging drops with a 500 μL reservoir volume. The best crystals were obtained from 4 μL hanging drops in which 2 μL of 10 mg mL^−1^ SeMet-Pex15 was mixed with 2 μL of a precipitant solution containing 5% PEG 6000 and 0.1 M MES pH 6.0, then streak-seeded with a cat whisker using previous crystals diluted and shattered by vortexing for 45 s. Crystals were harvested after soaking for 30 s in a cryoprotectant solution containing 5% PEG 6000, 0.1 M MES pH 6.0, and 25% 2-methyl-2,4-pentanediol.

Diffraction data for SeMet-Pex15 were collected at the ALS beamline 8.3.1 at Lawrence Berkeley National Laboratory. Data were collected at a temperature of ~100 K using a wavelength of 0.979560 Å after a selenium element scan of the crystals. The datasets were processed in the *P*2_1_ 2_1_ 2_1_ space group using XDS^66^ and the structure of SeMet-Pex15 was solved using Autosol^67^, from Phenix^68^. The structure was further refined with a 1.55 Å resolution cutoff using Phenix and Coot^69^ to an *R*_work_/*R*_free_ of 0.1734/0.1881. The crystal structure of Pex15 (43–253) includes the 6-residue PreScission Protease cleavage remnant LEVLFQ, which crystallized as a helix extending off of the last helix in the Pex15 structure. These six amino acids have been omitted from the structure shown in Fig. 1.

### Negative-stain EM

Twenty nanomolar of Pex1-FLAG/His-Pex6 complex was mixed with 200 nM Pex15 and incubated on ice for 5 min in the presence of 5 mM ATP. The sample was then diluted 100-fold with GF buffer without glycerol and containing 5 mM ATP, and 4 µL of the sample was applied to freshly plasma-cleaned 400 mesh Cu–Rh maxtaform grids (Electron Microscopy Sciences) that was coated with a thin layer of carbon. Prior to application of the sample on the grid, the carbon film was pre-treated with 0.1% (w/v) poly-l-lysine hydrobromide (Polysciences) to overcome preferred orientation of the complex on the carbon surface. These steps along with negative staining with 2% (w/v) uranyl formate were carried out as described in Gardner et al.^18^.

Negative stain data was acquired on a Tecnai Spirit (Thermo Fisher FEI) transmission electron microscope, operating at 120 kV, using the Leginon automated data acquisition system^70^. Micrographs were acquired at a nominal magnification of ×52,000 on an F416 CMOS 4K×4K camera (TVIPS), at a pixel size of 2.05 Å/pixel at the specimen level using a cumulative electron dose of 20 electrons/Å^2^, and a nominal defocus range between 0.5 and 1.5 µm.

A total of 1097 micrographs were collected for the complex. Initial steps of data processing and 2D analyses were performed using the Appion image processing pipeline^71^. The contrast transfer function (CTF) of each micrograph was determined using CTFFIND v3^72^ implemented within Appion. Particles were selected from micrographs using Difference of Gaussians (DoG)-based automated particle picker^73^. Phases for each micrograph were corrected using EMAN^74^. Particles were extracted using a 224 × 224 pixel box. Individual particles were normalized using the normalization function in the XMIPP package^75^ by eliminating pixels with values above or below 4.5*σ* of the mean pixel value. The particles were binned by a factor of two for faster computation. An initial stack of 102,101 particles was subjected to five rounds of iterative multivariate statistical analysis (MSA)^76^, and multi-reference alignment (MRA) in Appion to remove any erroneously picked non-particle features and aggregates, resulting in a final stack of 78,118 particles.

The Pex1/Pex6 map (EMD-6254) was low passed filtered to 60 Å resolution and used as a starting model for 25 iterations of three-dimensional classification in Relion v1.3^77^. 14,678 particles belonging to the best resolved three-dimensional class average of the full complex, out of ten classes, were subjected to three-dimensional refinement by projection matching in Relion. The final three-dimensional reconstruction (Fig. 1b) after convergence of refinement was at a resolution of 23.2 Å (Supplementary Figure 1C) by Gold Standard Fourier Shell Correlation, at a cutoff of 0.143. This final EM map was deposited in EM data bank (EMD-7005). All rigid-body fitting of atomic models into EM density, as well as generation of figures were performed using UCSF Chimera^78^.

### ATPase assays

Pex1/Pex6 ATPase activity was monitored using an ATP/NADH coupled enzyme assay in which the regeneration of hydrolyzed ATP is coupled to the oxidation of NADH^79^. The reaction contains 5 nM Pex1/Pex6, 3 U mL^−1^ pyruvate kinase, 3 U mL^−1^ lactate dehydrogenase, 1 mM NADH, 7.5 mM phosphoenolpyruvate, 2 μM BSA, and 5 mM ATP, with additional Pex15, Pex5, Ub-Pex5, or Ubp15. The absorbance of NADH was measured at 340 nm in a 96-well plate using a SpectraMAX 190 plate reader.

### Hydrogen deuterium exchange with mass spectrometry

For all HDX-MS experiments, the 100 μL exchange reactions were quenched by an equal volume of ice-cold quench buffer (400 mM KH_2_PO_4_/K_2_HPO_4_, pH 2.2) and the 200 μL samples were injected into an HPLC (Agilent 1100) system at a flow rate of 400 μL min^−1^ using 0.05% TFA as a mobile phase. Peptic peptides were generated in-line with digestion on two homemade columns (2 mm × 2 cm) with immobilized pepsin. The peptides resulting from inline-digestion were trapped on the reversed-phase column (1 mm × 2 cm; POROS 50 R2 material) and desalted for 3 min. After desalting, the peptides were eluted from the trap column over a 0.5 mm × 5 cm BioBasic-8 analytical reversed-phase column directly into the Orbitrap Discovery (Thermo) ESI source. The elution gradient profile was 15–55%B in 10 min, 55–100%B in 1 min, and then 100-15%B in 1 min at a flow rate of 17.5 μL min^−1^ in which buffer A is 0.05% TFA and buffer B is 90% acetonitrile, 0.05% TFA. To minimize back exchange, the system, including all columns, was immersed in an icebath. We extensively cleaned the HPLC system between runs and periodically ran blanks runs to mitigate peptide carryover. We note that the 100% deuterated control samples were always run last, so the appearance of the more deuterated, unfolded peaks are not due to carryover. Typical ESI source conditions used were: positive ionization mode, spray voltage 5.0 kV, capillary temperature 275 °C, nitrogen sheath gas 30 (arb) and capillary voltage/tube lens voltage 11/120 V. Collision-induced dissociation (CID) was carried out by selecting the 10 most intense ions from a high resolution (30,000) MS1 scan and subjecting them to the ion trap MS2 scan with following typical conditions: collision energy between 35 activation (*Q*), 0.25 V, and activation time 30 ms. Mass range was between 300 and 2000 *m*/*z*. The mass spectrometer was calibrated in positive mode using Pierce LTQ ESI positive ion calibration solution. Mass accuracy was 10 ppm.

Peptides from Pex1/Pex6, Pex15, and Pex5 were initially identified by running tandem MS/MS experiments of each protein alone. Peptides were identified using Proteome Discoverer 2.1 (Thermo). Data analysis was performed using HD-Examiner software (Sierra Analytics), followed by manual inspection of every peptide to check retention time, charge state, *m*/*z* range and the presence of overlapping peptides. The level of deuteration incorporation was calculated based on a non-deuterated and 100% deuterated control samples to account for back-exchange during sample preparation. For peptides without a 100% control, we estimated the back exchange to be 20%. Fully deuterated control samples were prepared by three cycles of drying and resolubilization in D_2_O buffer with 1 mM TCEP and 6 M guanidine HCl. For all samples in which we calculated a relative deuteration level, the relative deuteration levels were calculated using HDExaminer on a unimodal distribution.

To determine the Pex15-binding site on Pex1/Pex6, 60 μM Pex15 was incubated with 2 μM Pex1-WB2/Pex6-WB2 hexamer for 1 min. The amide hydrogen exchange was initiated by diluting the 5 μL reactions with 95 μL D_2_O buffer (60 mM HEPES pD 7.6, 50 mM NaCl, 50 mM KCl, 10% glycerol, 10 mM MgCl_2_, 0.5 mM EDTA, 5 mM ATP). The amide hydrogen exchange reaction consisted of 3 μM Pex15 1–309 and 100 nM Pex1/Pex6 and was quenched after 15 or 600 s. The relative deuteration level of Pex6 peptides with a score >0.85 were compared between the samples in the presence and absence of Pex15 using HDExaminer. We observed 65% protein coverage of Pex6.

In Pex1/Pex6 containing samples, an undigested fragment eluted after the Pex1 and Pex6 peptides, and its undeuterated mass was estimated using Thermo Excalibur ProMass to be 21978.2 Da, nearly the mass of 6XHis-Pex6 2-195 (the sequence GSSHHHHHHSQDPMKASLTFSLSGIYAPCSISRDIYLEYGDKKAECLYGTIRLPQYGPGCTPGKIVHCQVLDDSLPFCSIVVPSKLFGFMPTQPTMDFCYFEPILDNVVPVLDSVTFLINEQLYSKLMDLPQEMQQIQFLHYKYNINSMETVVHSRDILTSGLCQILNCSPFPQGLVDFTETQLILVNDTEQKLSA is 21,979 Da). We used Thermo Excalibur ProMass to calculate the molecular weight of this fragment in the presence and absence of Pex15 after deuteration for 0, 15, and 600 s (Supplementary Table 2).

For experiments shown in Fig. 3a and Supplementary Figure 7, we incubated 30 μM Pex15 1-309 with 2 μM Pex1/Pex6 hexamer or buffer for 60 s at 30 °C with 30 mM ATP. Amide hydrogen exchange was initiated by a 20-fold dilution into D_2_O buffer for 15 s, and the exchange reaction was quenched and the samples were analyzed as described above. For Fig. 3b, we incubated 30 μM Pex15 1-309 in buffer or with 2 μM Pex1/Pex6 hexamer for 5 s at 30 °C with 30 mM ATP before a 30 s deuteration. Using HDExaminer, we calculated the relative deuteration level of selected peptides compared to not deuterated or 100% deuterated controls and mapped the deuteration level by color on the crystal structure of Pex15’s core domain. The peptide spectra are shown in Supplementary Figure 5. Note that not all peptides are mapped onto the core domain structure, but the relative deuteration levels are also shown in Supplementary Figure 1A. For experiments shown in Fig. 3c, we incubated 10 μM MBP-Pex15 1-309 with 2 μM Pex1/Pex6 or buffer for 60 s at 30 °C with 6 mM ATP. After the 60 s incubation, the samples were diluted 20-fold into D_2_O buffer, quenched after 15 s of deuteration and analyzed as described above.

In Fig. 4, we combined 5 mM ATP and 100 nM Pex1/Pex6 (WT or WB2) in 99 μL D_2_O buffer (60 mM HEPES pH 7.6, 50 mM NaCl, 50 mM KCl, 10% glycerol, 10 mM MgCl_2_, 0.5 mM EDTA), and initiated Pex15 unfolding and amide hydrogen exchange simultaneously by the addition of 1 μL of 150 μM Pex15 1-309, for a final concentration of 1.5 μM Pex15. The exchange reaction was quenched at various time points with ice-cold quench buffer (400 mM KH_2_PO_4_/K_2_HPO_4_, pH 2.2), and analyzed as previously stated.

For analysis of the EX1 kinetics of Pex15 peptides, we calculated the percent of the total in the folded and unfolded population of two representative peptides (aa 71–85 and aa 216–234) by fitting the curves with a double Gaussian and plotting over time^80^. We used the first two points to estimate the initial linear rate of Pex15 unfolding by Pex1/Pex6 at 60 Pex15 unfolded per Pex1/Pex6 hexamer per minute for peptide 1 (aa 71–85) and 42 Pex15 unfolded per Pex1/Pex6 hexamer per minute for peptide 2 (aa 216–234).

For Supplementary Figure 10, we looked for changes in Pex5 amide hydrogen exchange in the presence and absence of Pex1/Pex6, Pex15 1-309, and MBP-Pex15 1-327. Pex5 and Pex15 were pre-incubated together, and then deuteration and potential unfolding were initiated simultaneously by the addition of 43 nM Pex1/Pex6, 3 mM ATP in D_2_O buffer. The exchange reaction with final concentrations of 1.2 μM Pex5, 1.5 μM Pex15, and 43 nM Pex1/Pex6 was quenched after 20 s at 30 °C .

### Maleimide-labeling of Pex15

Maleimide-labeling reactions were performed using fluorescein-5-maleimide (Anaspec Inc.). Substrate proteins were used at a final concentration of 2.5 μM, and each reaction included an ATP regeneration mixture (ATP, creatine kinase, creatine phosphate). Substrates were diluted using Buffer1 (50 mM HEPES, 50 mM NaCl, 50 mM KCl, 10 mM MgCl_2_, pH 7.5) before the addition of Pex1/Pex6 complex (*C*_f_= 0.1–0.2 μM). The reaction was incubated at the designated temperature for 60 s with the motor for unfolding, then for an additional 30 s with fluorescein maleimide (*C*_f_=50 mM) before quenching with equal volume quench buffer (2% SDS, 10% β-mercaptoethanol). “Urea” samples were diluted in Buffer2 (50 mM HEPES, 50 mM NaCl, 50 mM KCl, 10 mM MgCl_2_, 8 M urea, pH 7.5) to a 6 M urea final concentration and incubated with F5M at the designated temperature for 10 min before quenching. Excess F5M was run off 10% or 12% acrylamide SDS-PAGE gels, and the gels were imaged in the fluorescein channel (Bio-Rad ChemiDoc MP Imaging System) prior to Coomassie staining.

To quantify the percent of unfolded Pex15 by maleimide labeling, each reaction was run in triplicate and the SDS-PAGE gels were imaged on a Typhoon Trio Variable Mode Imager using the Typhoon Scanner Control v5.0 software (GE Healthcare). Gels in a single experiment were scanned simultaneously in a single channel 526 SP/Blue (488 nm) with a pixel size of 50 μm. The resulting gel images were quantified using ImageQuant TL’s 1D gel analysis toolbox (GE Healthcare Life Sciences). Gel lanes were adjusted to exclude signal from adjacent lanes. Bands corresponding to Pex15 were manually selected using ImageQuant’s automatic detection peak boundaries and snap to peak editing. Background signal was subtracted using the rolling ball subtraction. The peak signal volume, excluding background signal, was used for quantification. For each Pex15 construct, the percent unfolded was calculated by subtracting the basal fluorescence signal from the signal of all samples within each replicate of a given construct. The signals for each sample were then averaged, and the percent unfolded calculated by setting basal fluorescence to 0% and urea-containing sample fluorescence to 100%.

### Pull-down binding assays of Pex1/Pex6 with Pex15

Pull-down binding assays with Pex1-Strep/His-Pex6 WT and pore-loop mutants were performed in 50 μL reactions containing 1.2 μM Pex1/Pex6, 21 μM Pex15 1-327-FLAG-His, and ATP regeneration mix. Each reaction was incubated with beads from 30 μL of slurry of IBA MagStrep magnetic beads and washed with 4 × 1 mL with 1 mL of 60 mM HEPES pH 7.6, 150 mM NaCl, 150 mM KCl, 10% glycerol, 0.05% Tween-20, 10 mM MgCl_2_, 0.5 mM EDTA, and 1 mM ATP. Pex1-Strep and bound proteins were eluted with 2 mM biotin.

### Pull-down binding assays of Pex1/Pex6 with GST-Pex14

Pull-down binding assays with GST-Pex14 were performed in 60 μL reactions containing 4 μM GST-Pex14, 1.7 μL FLAG-Pex5-His, 2 μM Pex15 1-327-FLAG-His, 0.5 μM Pex1/Pex6 hexamer, and ATP regeneration mix. Each reaction was incubated with 15 μL of slurry of glutathione magnetic agarose beads (Pierce) and washed with 4 × 1 mL washes of 60 mM HEPES pH 7.6, 150 mM NaCl, 150 mM KCl, 10% glycerol, 10 mM MgCl_2_, 0.5 mM EDTA, 1 mM ATP, 0.05% Tween-20, and 0.1% Triton X-100. GST-Pex14 and bound proteins were eluted with 20 mM glutathione. FLAG-tagged Pex15 was detected by Western blot with an anti-FLAG M2 HRP-conjugated antibody (Sigma A8592).

### Pull-down binding assays of MBP-Pex15 and Pex5

Two micromolars of MBP-Pex15 1-327 was incubated with 350 nM Pex5-FAM, 1 μM Pex1-WB2/Pex6-WB2, and 100 μM SKL peptide and ATP in 50 μL reactions with magnetic amylose resin (New England BioLabs) for 1 h in 60 mM HEPES pH 7.6, 150 mM NaCl, 150 mM KCl, 10% glycerol, 0.05% Tween-20, 10 mM MgCl_2_, 0.5 mM EDTA, and 5 mM ATP at 4 °C. The magnetic beads were washed four times with 1 mL of the same buffer, and eluted in buffer+20 mM maltose. Proteins from the input and elution were separated by SDS-PAGE and visualized by fluorescein on the Typhoon prior to Coomassie staining.

### Pull-down binding assays with Pex6-N1 domain

One micromolar of MBP-Pex15 1-327-FLAG-His was incubated alone or with 0.5 μM Pex1-Strep/His-Pex6 hexamer, His-Pex1-FLAG, His-Pex6-FLAG, or 5 μM His-Pex6 1-215 and magnetic amylose resin (New England BioLabs) for 1 h in 60 mM HEPES pH 7.6, 150 mM NaCl, 150 mM KCl, 10% glycerol, 0.05% Tween-20, 10 mM MgCl_2_, 0.5 mM EDTA+5 mM ATP at 4 °C. The magnetic beads were washed four times with 1 mL of the same buffer, and eluted in buffer+20 mM maltose. Proteins from the input and elution were separated by SDS-PAGE and either visualized by SYPRO-Ruby on the Typhoon or transferred to a PVDF membrane for detection by western blot with an anti-His HRP-conjugated antibody (ThermoFisher PA1-23024).

### Peroxisome matrix-protein import assays

All yeast strains were constructed in a background of W303 *MATa ura3-1 his3-11 trp1-1 leu2-3 leu2-112 can1-100*. Individual knockout strains for *pex1Δ::NatR, pex6Δ::NatR*, and *pex15Δ::KanR* were constructed using standard transformation techniques and the pFA6A-NatMX or KanMX plasmids.

The violacein pathway (VioA, VioB, and VioE-SKL) was integrated into the knockout strains at the *leu2* locus by digesting pWCD1401 or pWCD1402 with NotI-HF, gel extraction of the 11.5 kB fragment, subsequent transformation into yeast and selection on SD-Leu media^56^.

To complement the *pex1Δ*, *pex6Δ*, or *pex15Δ* gene deletions, we incorporated a genomic fragment containing the ORF and endogenous promoter and terminator into the pRS316 plasmid backbone using NotI and SalI: Pex1 ORF with 300 nucleotides upstream, 350 nucleotides downstream; Pex6 ORF with 200 nucleotides upstream, 148 nucleotides downstream; and Pex15 ORF with 352 nucleotides upstream, 150 nucleotides downstream. Mutant alleles of Pex1 and Pex6 were incorporated by Gibson cloning combining a PCR containing the Pex1 or Pex6 ORF from *E. coli* expression plasmids and a PCR of the pRS316 vector backbone containing the promoter and terminator regions. Truncations of the N-terminus of Pex15 were made by around the horn PCR of the vector backbone and blunt-end ligation of the phosphorylated ends.

The recovering plasmids were transformed into the knockout, violacein-pathway containing yeast strains and selected on SD-Ura-Leu. Three colonies from each transformation were streaked for singles on SD-Ura-Leu plates, then a single colony from each was picked for growth overnight, dilution and growth to log-phase the next day, and spotted with 5 μL at OD_600_ = 0.3.

### Data availability

Crystallographic coordinates have been deposited in the RCSB with accession code 5VXV. Electron microscopy density has been deposited in the EMDB with accession code 7005. The datasets generated during and/or analyzed in the current study and other data are available from the corresponding author on reasonable request.

## Electronic supplementary material

Supplementary Information
