# Supplementary material for: The peroxisomal AAA-ATPase Pex1/Pex6 unfolds substrates by processive threading
Source: Nat Commun. 2018 Jan 10;9:135. doi: 10.1038/s41467-017-02474-4 (PMC5762779; doi:10.1038/s41467-017-02474-4)
Supplement: Supplementary file 1 — Supplementary Information [file 41467_2017_2474_MOESM1_ESM.pdf]

Supplementary Figure 1

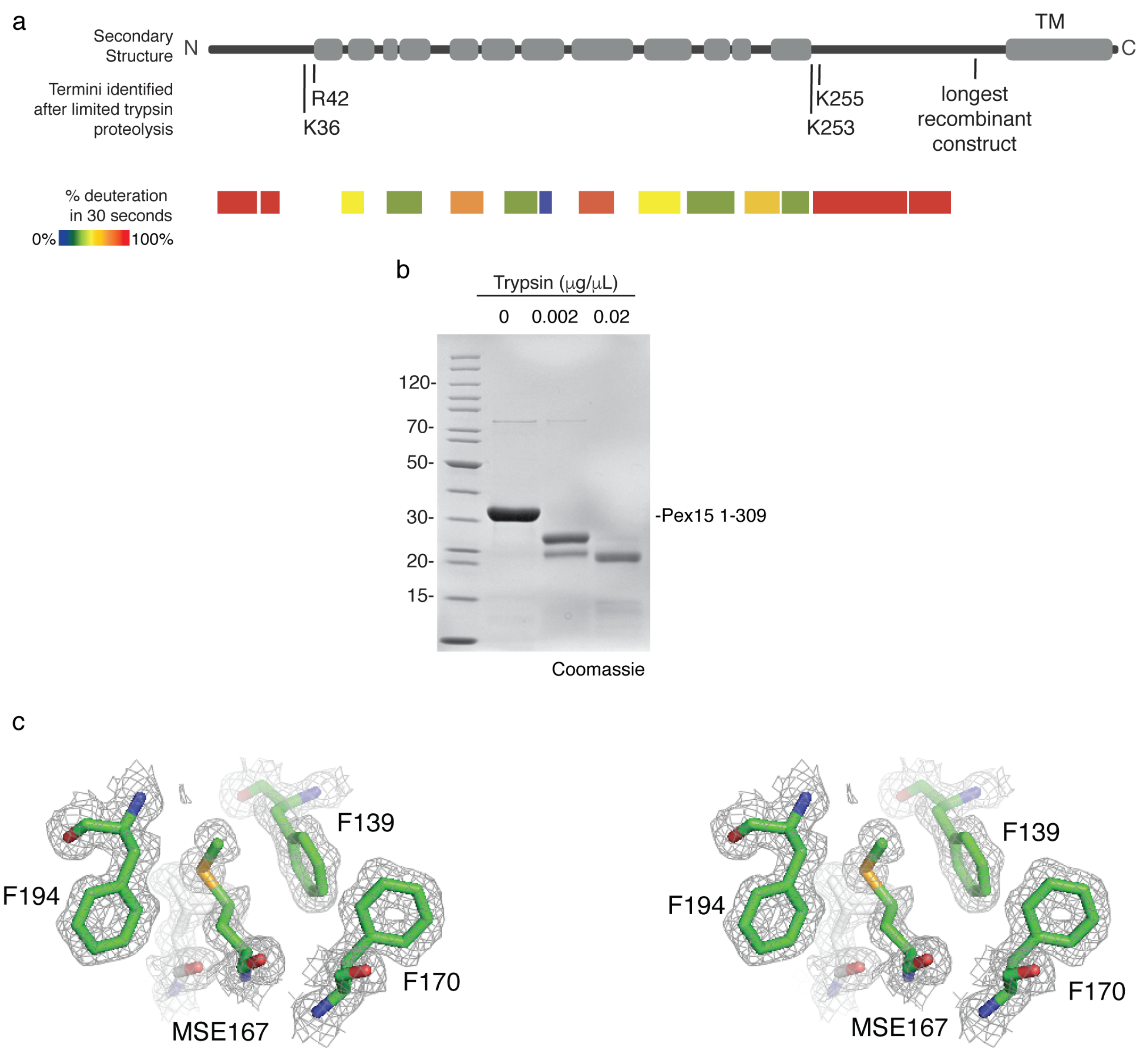

Supplementary Figure 1. The Pex15 cytosolic domain has a folded core and disordered N- and C-terminal segments. a) A cartoon model of Pex15 illustrates the α-helices in the core domain (gray bars) as determined by X-ray crystallography as well as the predicted helix for the transmembrane domain that was not part of the recombinant construct. Aligned underneath are the relative deuteration levels (colored bars) of selected peptides (72% coverage) for Pex15 after 30 seconds of deuteration. See Figure 3b for mapping of these peptides onto the Pex15 core structure. b) The core domain of Pex15 was identified by limited proteolysis of Pex15 1-309, followed by mass spectrometry of the isolated fragments. The N- and C-termini of the proteolytically stable fragments are indicated in panel a. c) A stereoimage of the superposition of the refined Pex15 core structure on an *ab initio* electron density map at contour level $\sigma = 1$, with the Se of a selenomethionine residue used for experimental phase determination shown in orange.

Supplementary Figure 2

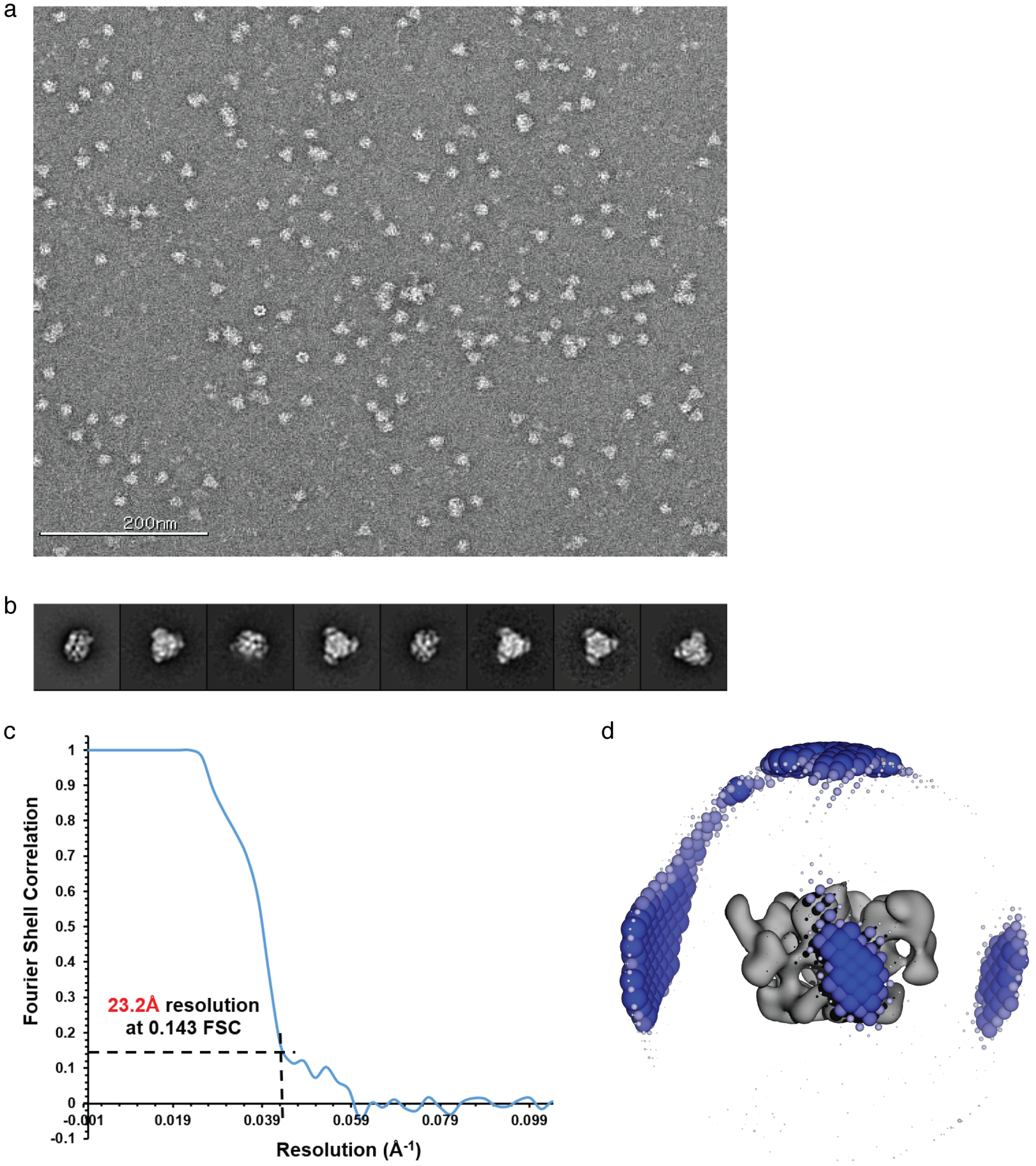

Supplementary Figure 2. Negative-stain EM of Pex15-bound Pex1/Pex6. a) Negative stained micrograph of Pex1/Pex6 in complex with Pex15. b) Representative 2D class averages showing different orientations of the complex in solution. c) Gold standard Fourier Shell Correlation (FSC) plot reporting the resolution of the final reconstruction to be 23.2 Å (at 0.143 FSC). d) Angular distribution of particles contributing to the 3D reconstruction, with the diameter of each sphere related to the number of particles at that Euler angle.

Supplementary Figure 3

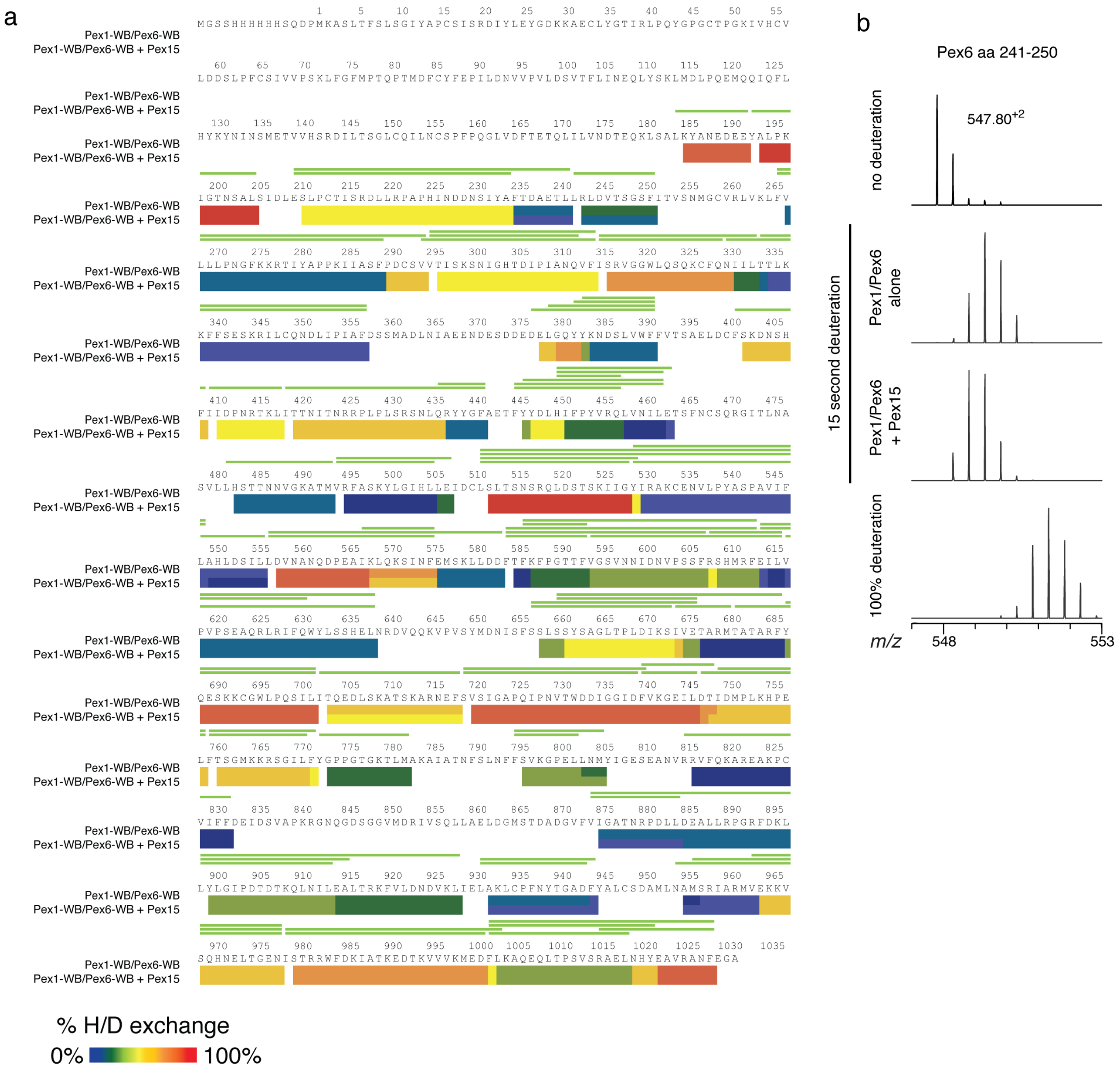

Supplementary Figure 3. Pex15 binding to Pex1/Pex6 protects a peptide in the N2 domain of Pex6 from solvent exchange. a) Peptide coverage of Pex6 (green bars, 65% coverage) and relative deuteration levels (rainbow bars) for the Pex1-WB2/Pex6-WB2 mutant hexamer after 15 seconds of deuteration in the absence and presence of saturating Pex15. b) Mass spectra of Pex6 aa 241-250 after 15 seconds of deuteration in isolation or with saturating Pex15. Control spectra of no deuteration and 100% deuteration are shown for comparison.

Supplementary Figure 4

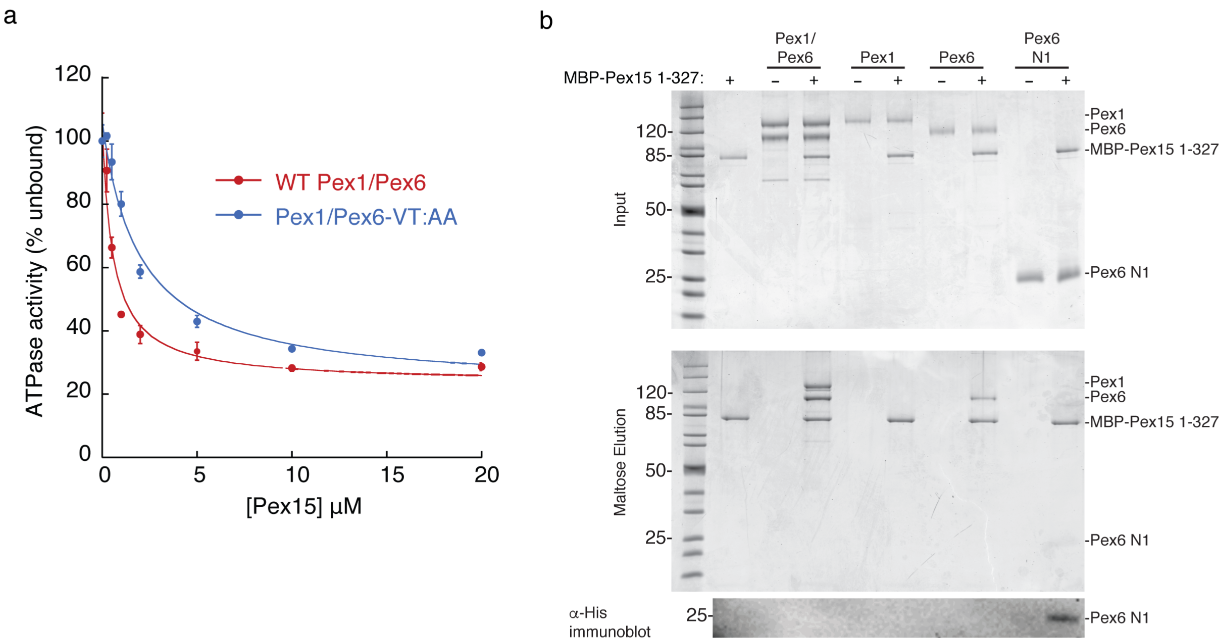

Supplementary Figure 4. Pex15 binds the Pex6 domains. a) Mutations in Pex6 (V245A, T246A) within the region that is protected in the presence of Pex15 (Supplementary Figure 3) alter the motor's binding affinity for Pex15, increasing the apparent K_D from 0.58 ± 0.18 µM for wild-type to 1.98 ± 0.58 µM for the mutant. ATPase data shown are the mean ± s.d. for n = 3 technical replicates. b) The Pex1/Pex6 hexamer, Pex6, and the Pex6 N1 domain pull down with MBP-Pex15 1-327, while Pex1 does not.

Supplementary Figure 5

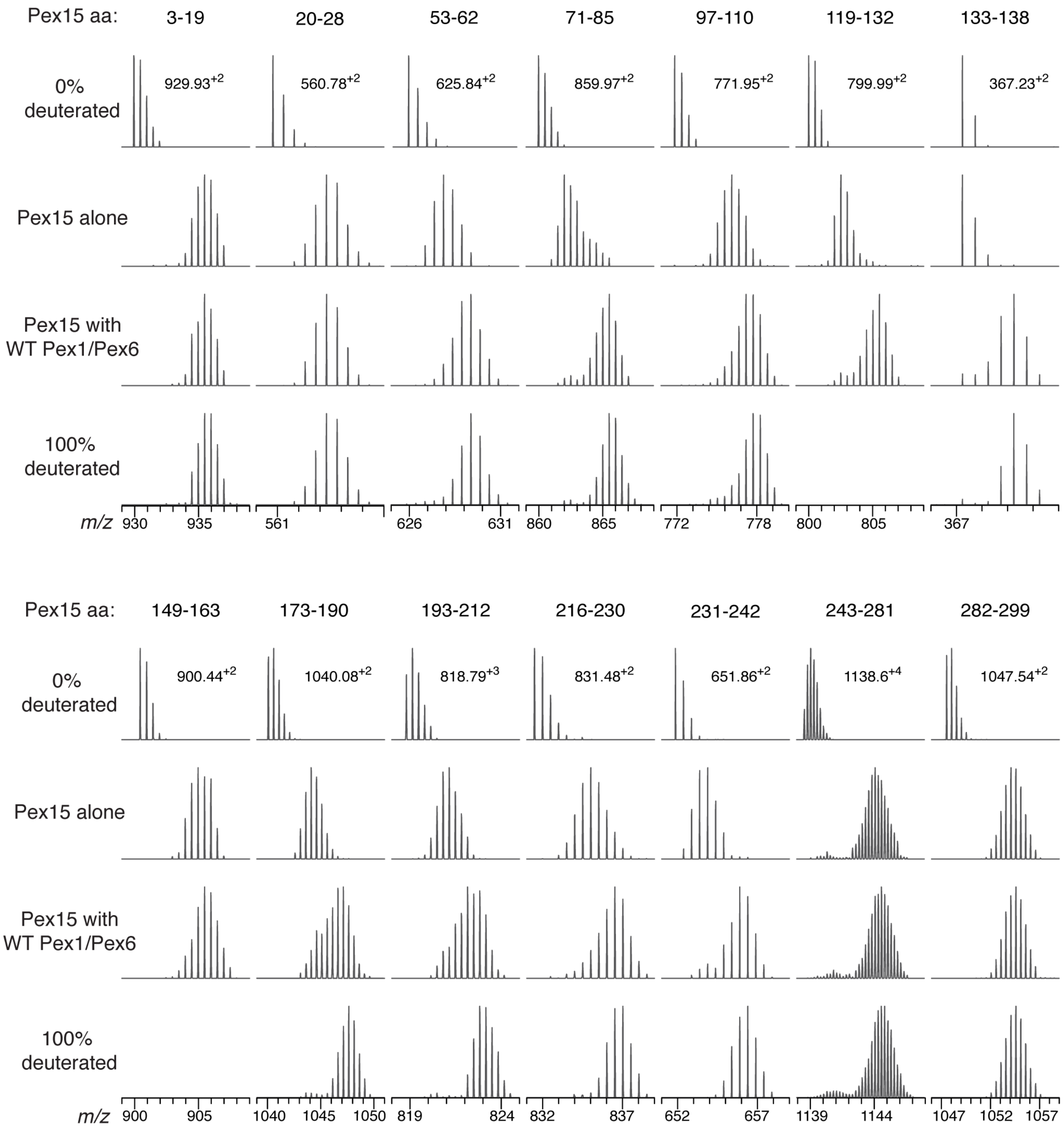

Supplementary Figure 5. Pex1/Pex6 unfolds Pex15. Mass spectra of Pex15 peptides mapped onto the structure in Fig. 3B. Pex15 1-309 in isolation or with 100 nM Pex1/Pex6 was deuterated for 30 seconds. Control spectra of no deuteration with the monoisotopic mass and charge and the spectra of the 100% deuterated peptide are shown for comparison. For peptides without a 100% deuterated spectra, the relative deuteration levels were calculated with the assumption of 20% back-exchange.

Supplementary Figure 6

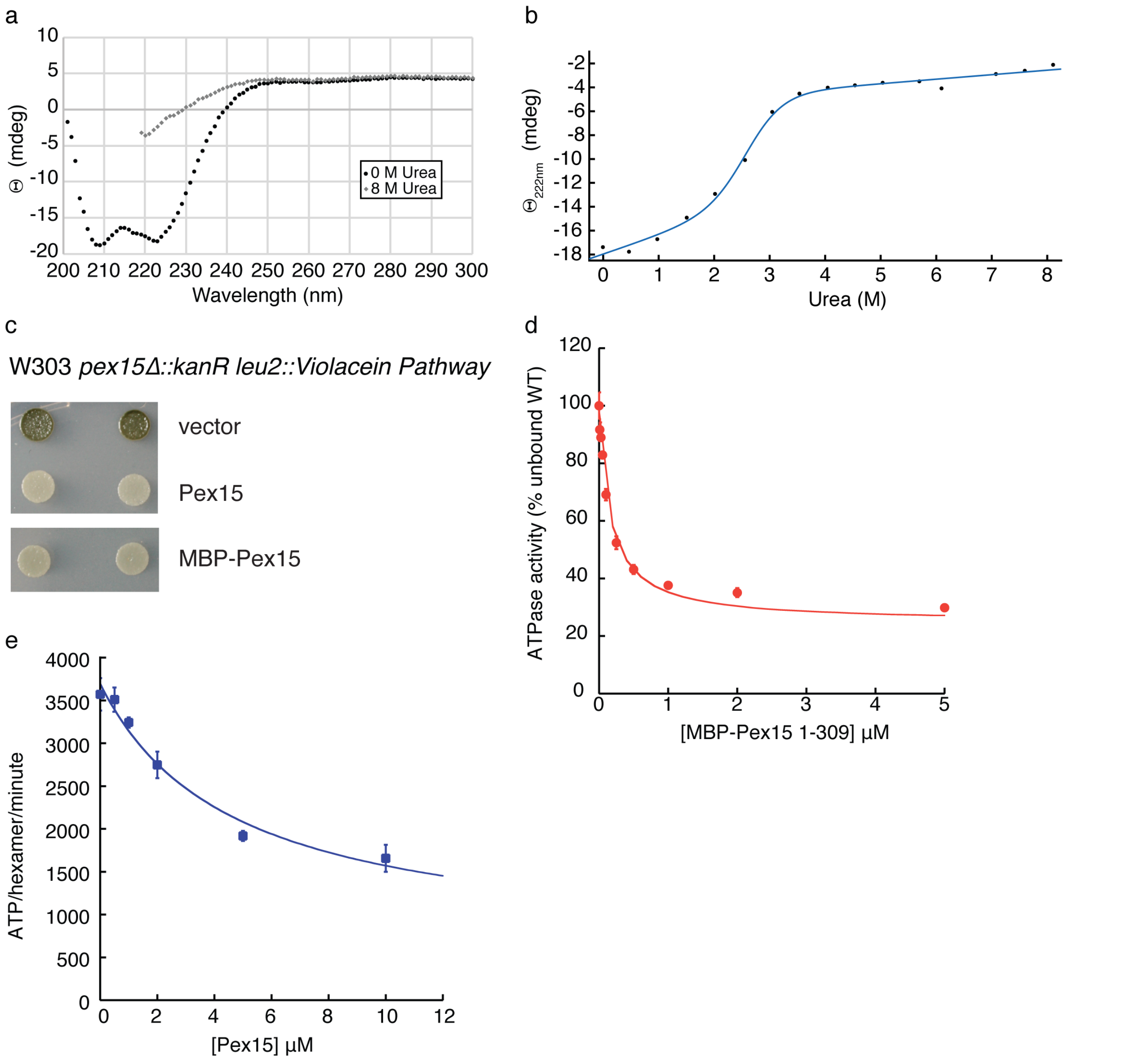

Supplementary Figure 6. Supporting data for interpreting Pex15 unfolding by Pex1/Pex6. a) The CD spectrum of native Pex15 1-309 at 30 °C shows a strong α-helical signal that is reduced for the denatured protein in 8 M urea. b) Urea-induced denaturation of Pex15 1-309 at 30 °C measured by the CD signal at 222 nm reveals a single cooperative unfolding event. c) The ability of the MBP-Pex15 fusion protein to support efficient peroxisomal matrix-protein import was assessed by colorimetric assay as in Figure 5b. d) MBP-Pex15 1-309 inhibits Pex1/Pex6 ATPase activity. ATPase data shown are the mean +/- s.d. for n = 3 technical replicates. e) Pex15 inhibits the ATPase activity of the Pex1/Pex6 sample used for the kinetic assays in Figure 4. At 1.5 µM Pex15 1-309, the motor uses approximately 3000 ATP per hexamer per minute. ATPase data shown are the mean +/- s.d. for n = 3 technical replicates.

Supplementary Figure 7

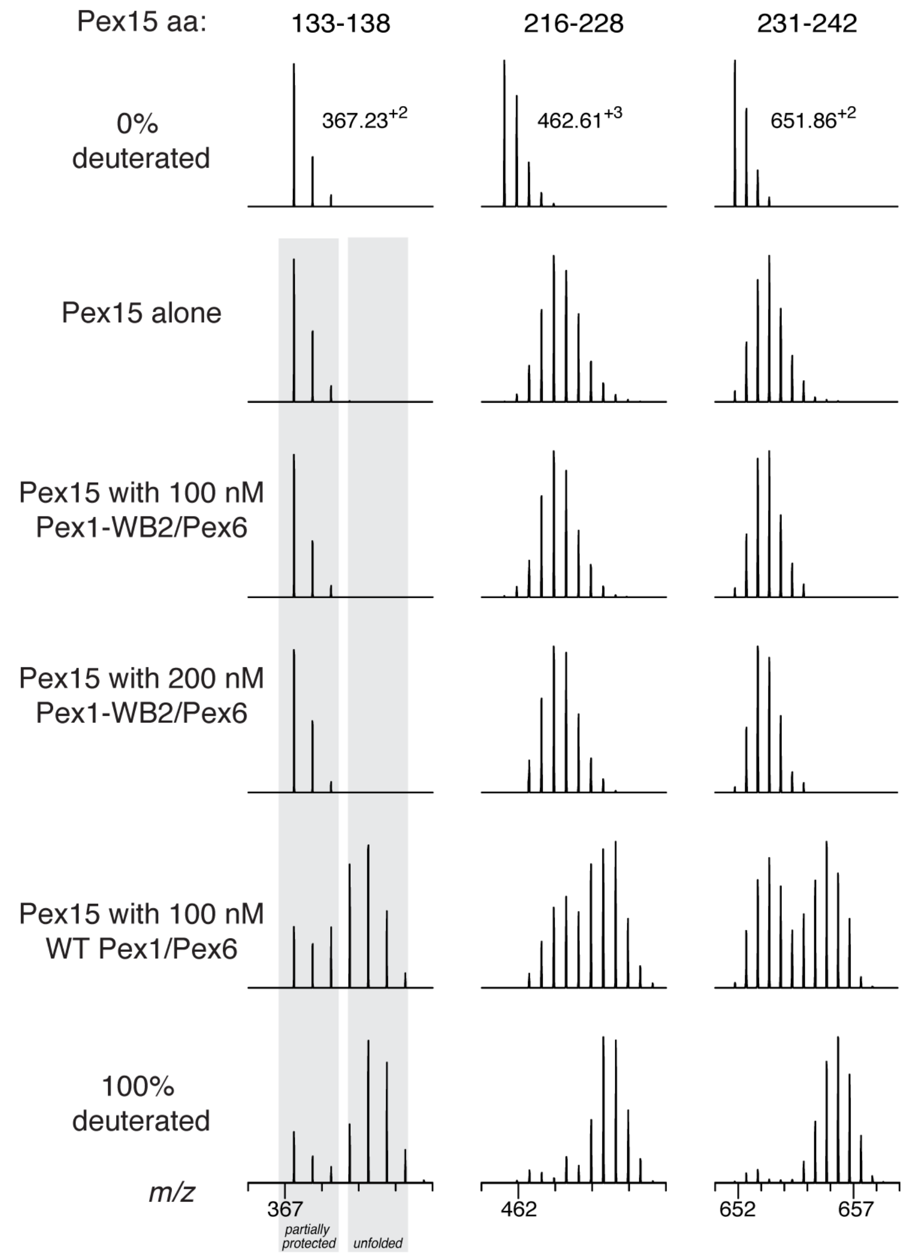

Supplementary Figure 7. Pex15 is not unfolded by Pex1/Pex6 with ATPase-deficient Pex1. Mass spectra of Pex15 peptides show no indication of Pex15 unfolding in the presence of 100 or 200 nM Pex1-WB2/Pex6, although Pex15 is unfolded in the presence of wild-type Pex1/Pex6. Control spectra with no deuteration and 100% deuteration are shown for comparison. Note that there was carry-over in the 100% deuteration control for this experiment.

Supplementary Figure 8

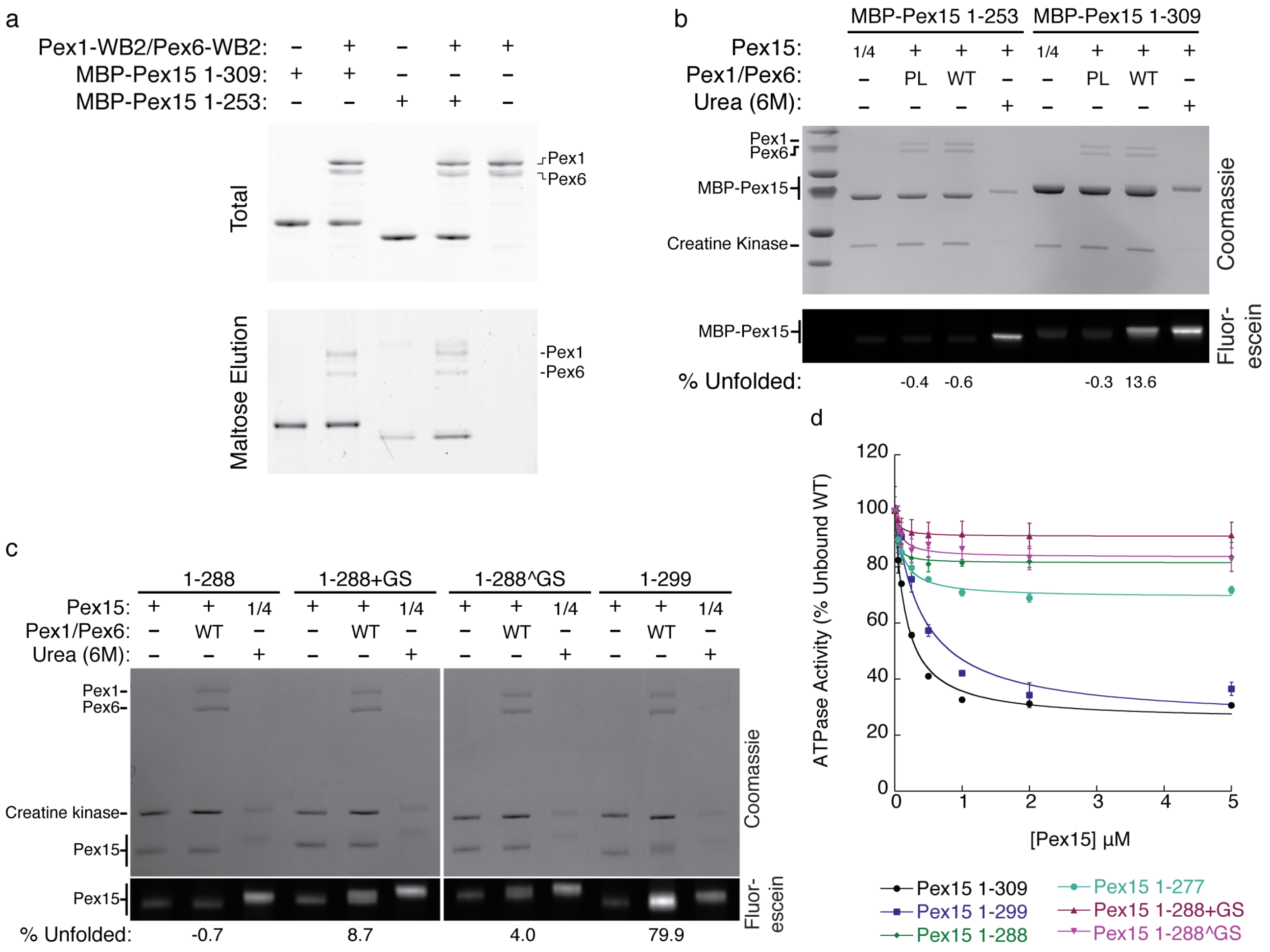

Supplementary Figure 8. Pex1/Pex6 mediated unfolding of Pex15 depends on a long C-terminal disordered segment. a) MBP-Pex15 1-253 and MBP-Pex15 1-309 pull down Pex1-WB2/Pex6-WB2 mutants with comparable affinity. b) Fluorescein-maleimide labeling of internal cysteines of MBP-Pex15 1-253 and MBP-Pex15 1-309 in isolation and in the presence of wild-type or pore-loop mutant Pex1/Pex6 (MBP does not contain any cysteine residues). The % unfolded in b and c are normalized to the levels of basal labeling or labeling in the presence of 6 M urea. c) Fluorescein-maleimide labeling of internal cysteines of Pex15 C-terminal truncation mutants with an additional Gly-Ser segment either inserted (Pex15 1-288^GS) or appended (Pex15 1-288+GS), alone and in the presence wild-type Pex1/Pex6. d) Inhibition of wild-type Pex1/Pex6 ATPase activity by the Pex15 C-terminal truncation constructs. ATPase data shown are the mean +/- s.d. for n = 3 technical replicates.

Supplementary Figure 9

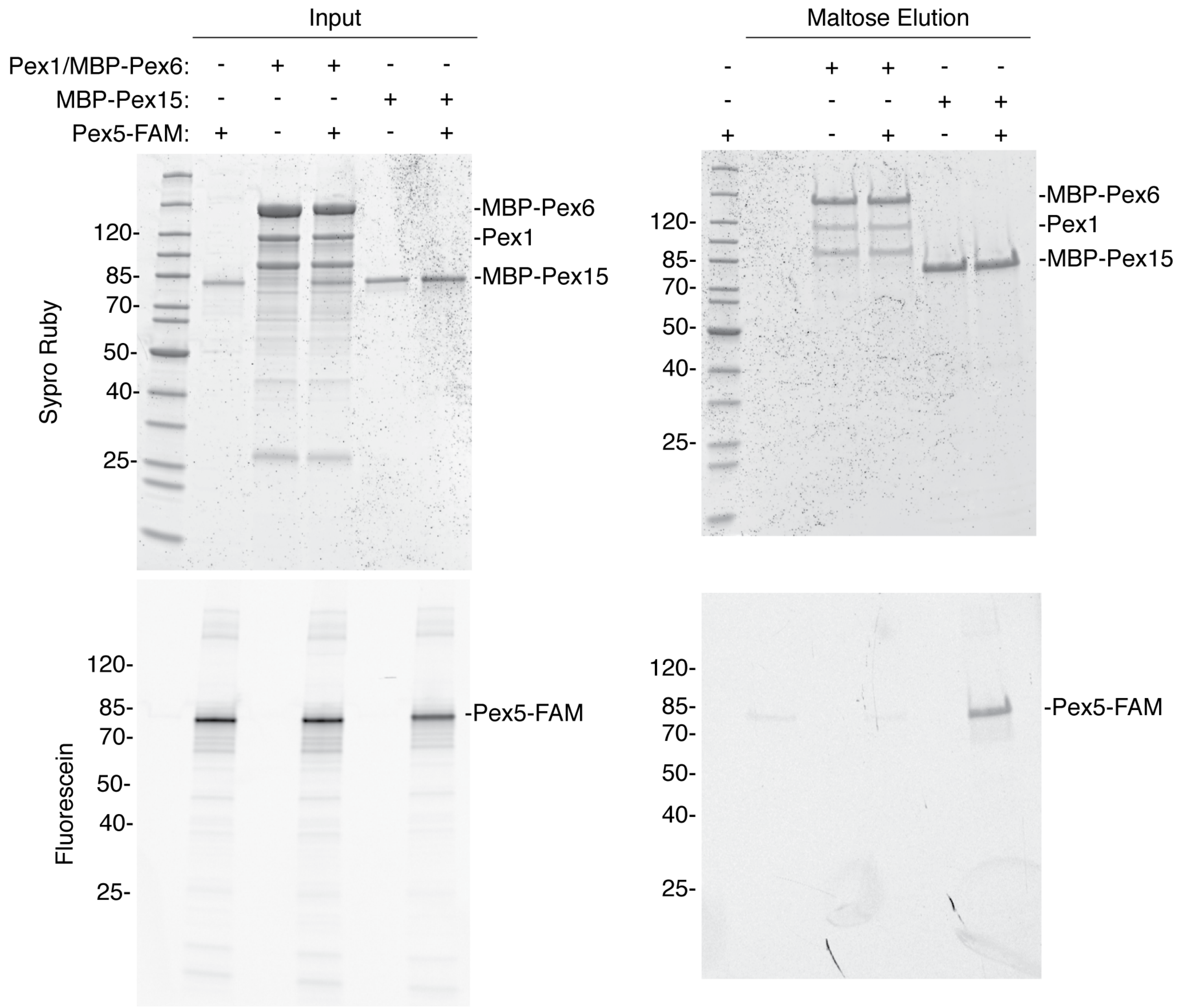

Supplementary Figure 9. Pex5 directly binds MBP-Pex15. Pull-down binding assay of MBP-Pex6/Pex1 and MBP-Pex15 1-327 with fluorescein labeled Pex5. Pex5 does not pull down with MBP-Pex6/Pex1, but does interact with MBP-Pex15 1-327. We note that there is a Pex1/Pex6 degradation product in the MBP-Pex6/Pex1 hexamer. MBP-Pex15 and Pex5-FAM do not resolve well by SDS-PAGE due to their similar molecular weights, but Pex5-FAM can be distinguished by the fluorescein label. This experiment was performed under the same conditions as Figure 7B.

Supplementary Figure 10

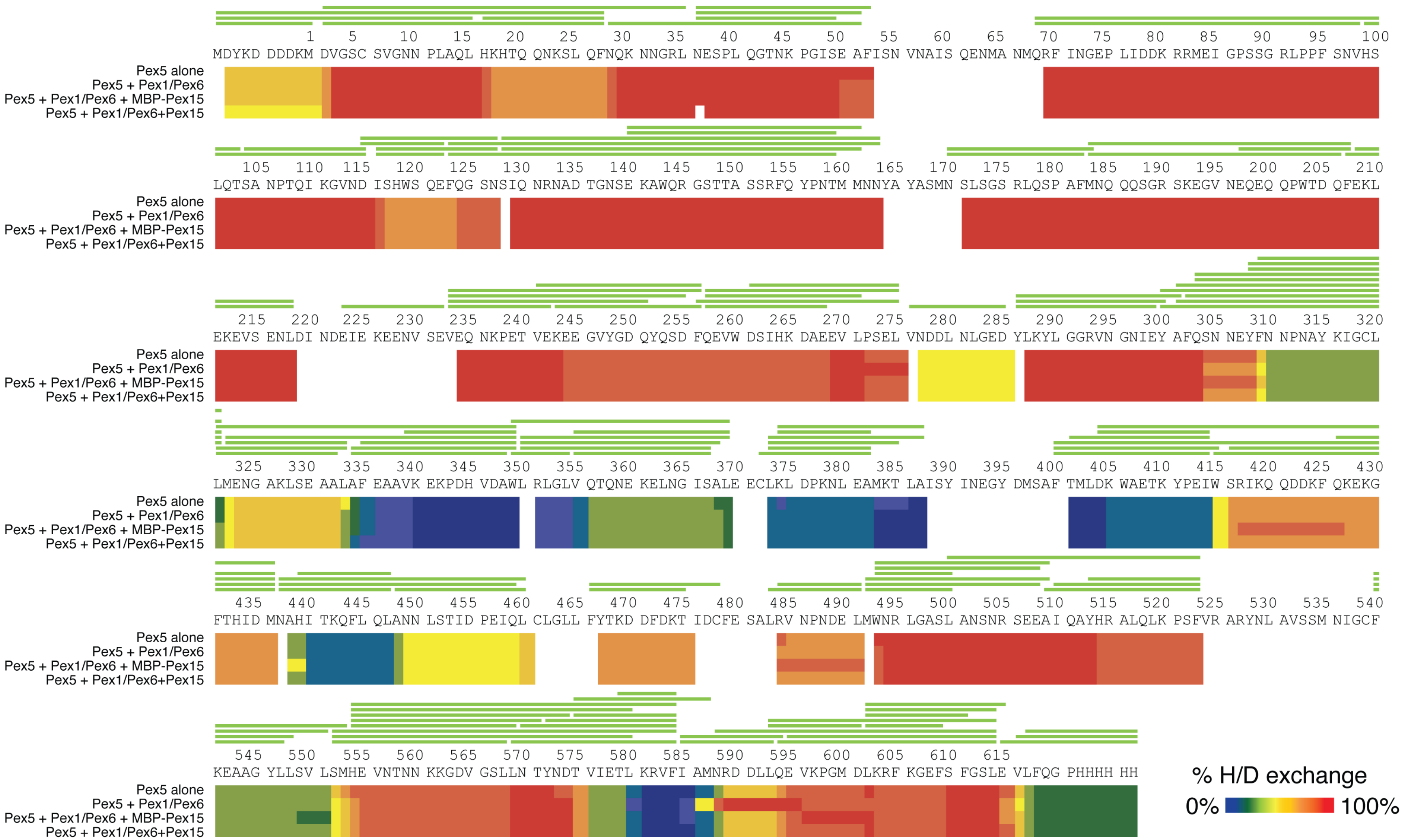

Supplementary Figure 10. Pex5 is not unfolded by Pex1/Pex6, even in the presence of Pex15.
Peptide coverage of Pex5 (green bars, 88% coverage) and relative deuteration levels (rainbow bars) in isolation and with Pex1/Pex6 hexamer, Pex15 1-309, and MBP-Pex15 1-327 after 20 second deuteration. The relative deuteration levels of peptides from Pex5 were not altered by incubation with Pex1/Pex6, Pex15 1-309 or MBP-Pex15 1-327.

Supplementary Figure 11

Uncropped gels for Figure 1b.

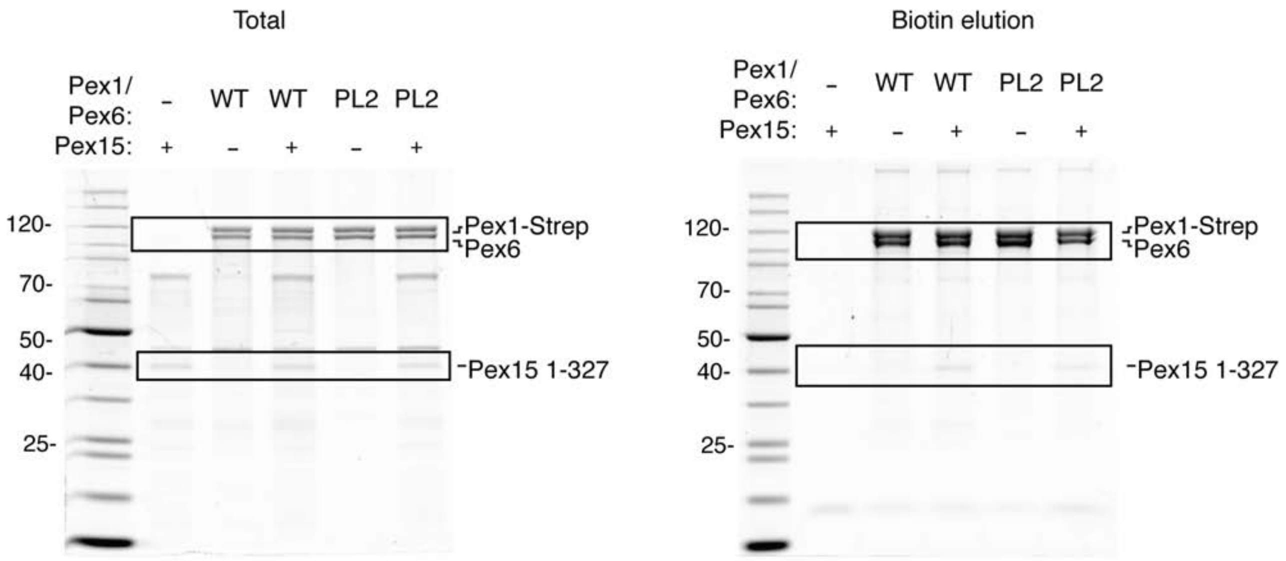

Uncropped gels for Figure 5a.

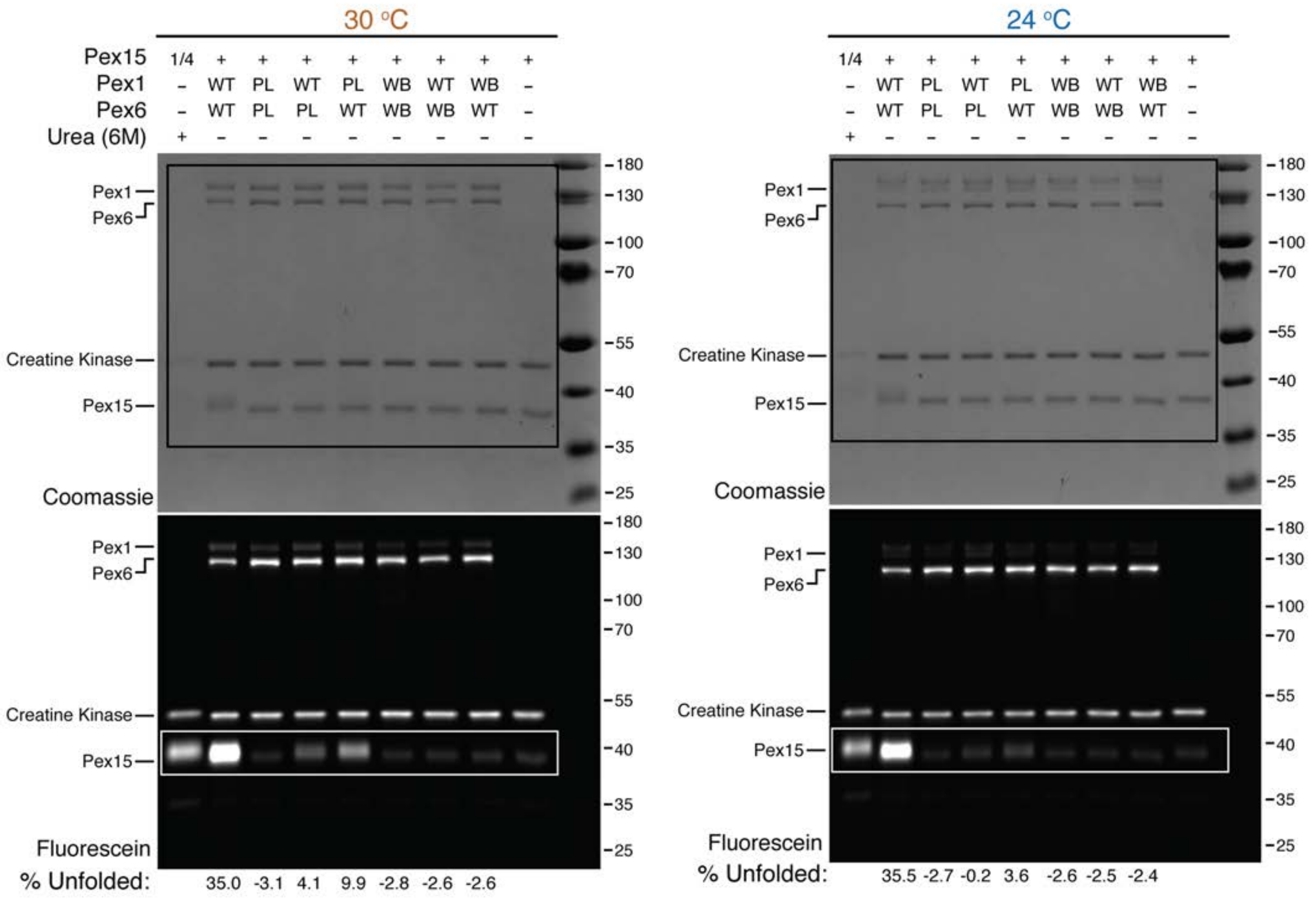

Uncropped gels for Figure 6c.

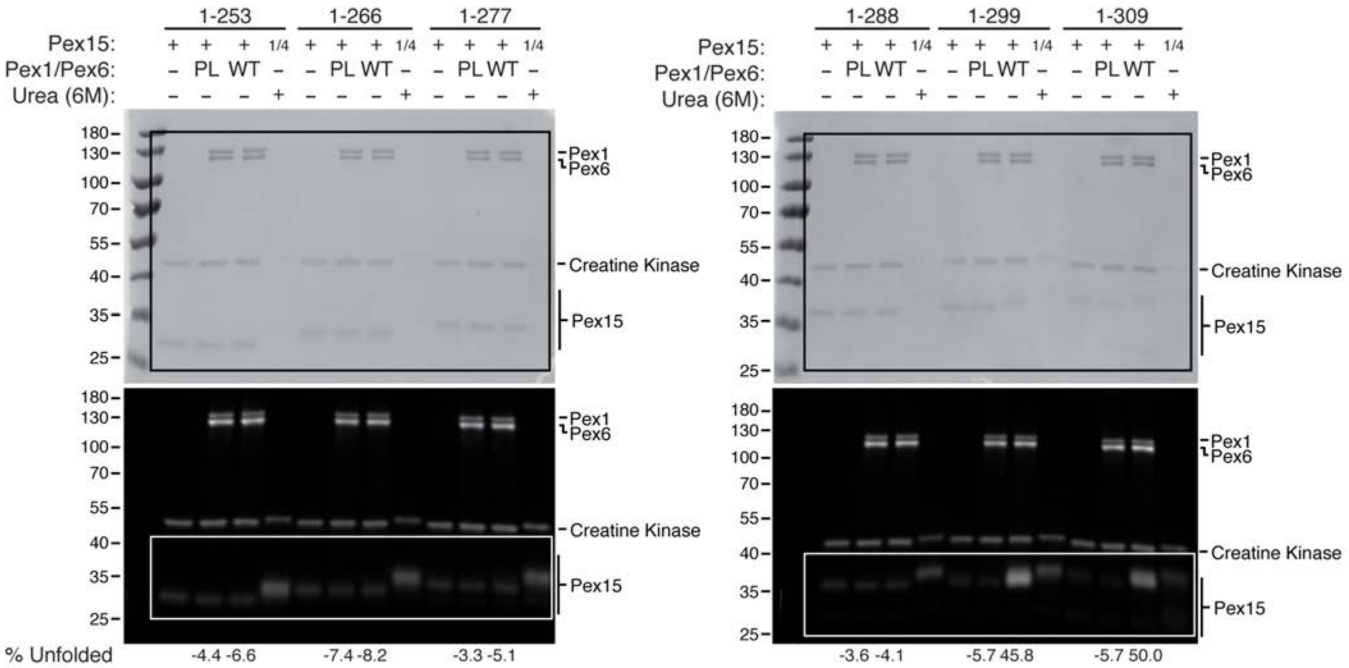

Uncropped gels for Figure 7a.

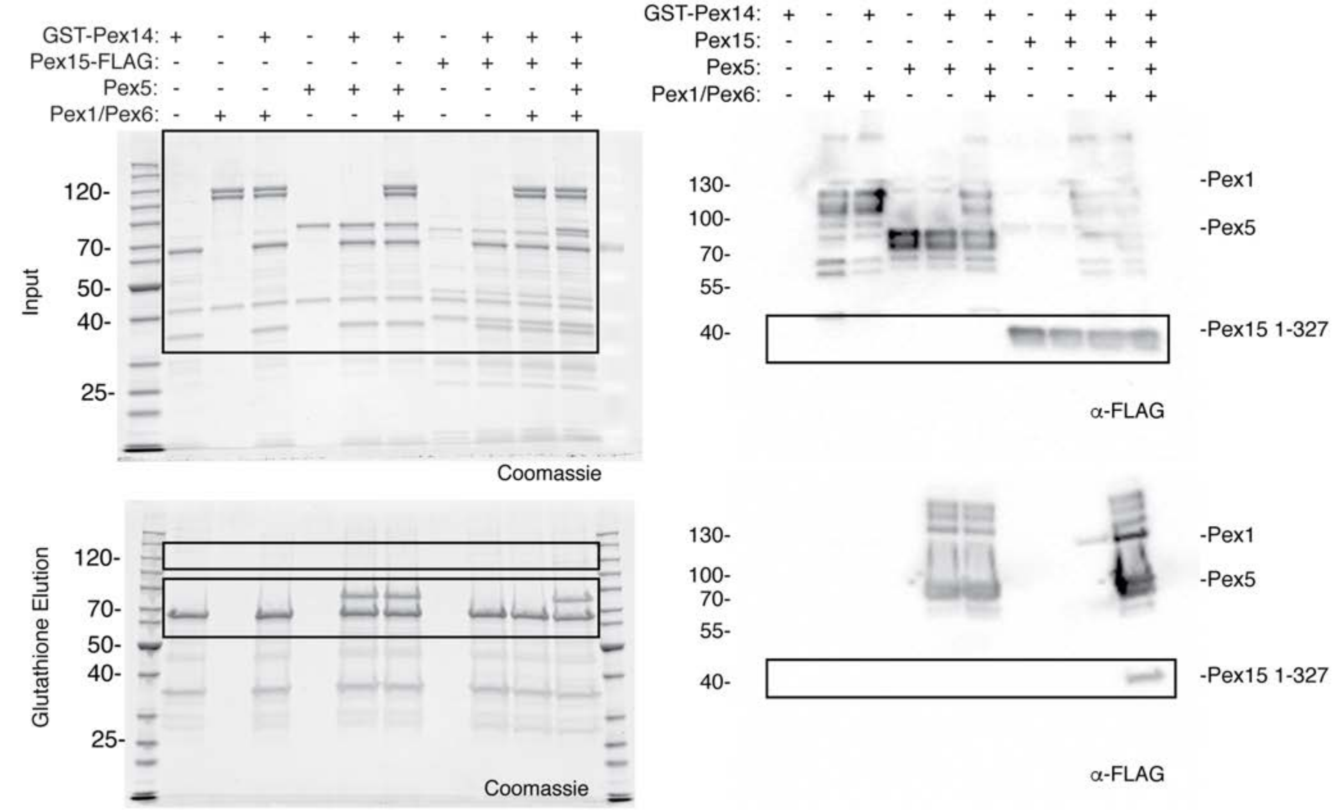

Uncropped gels for Figure 7b.

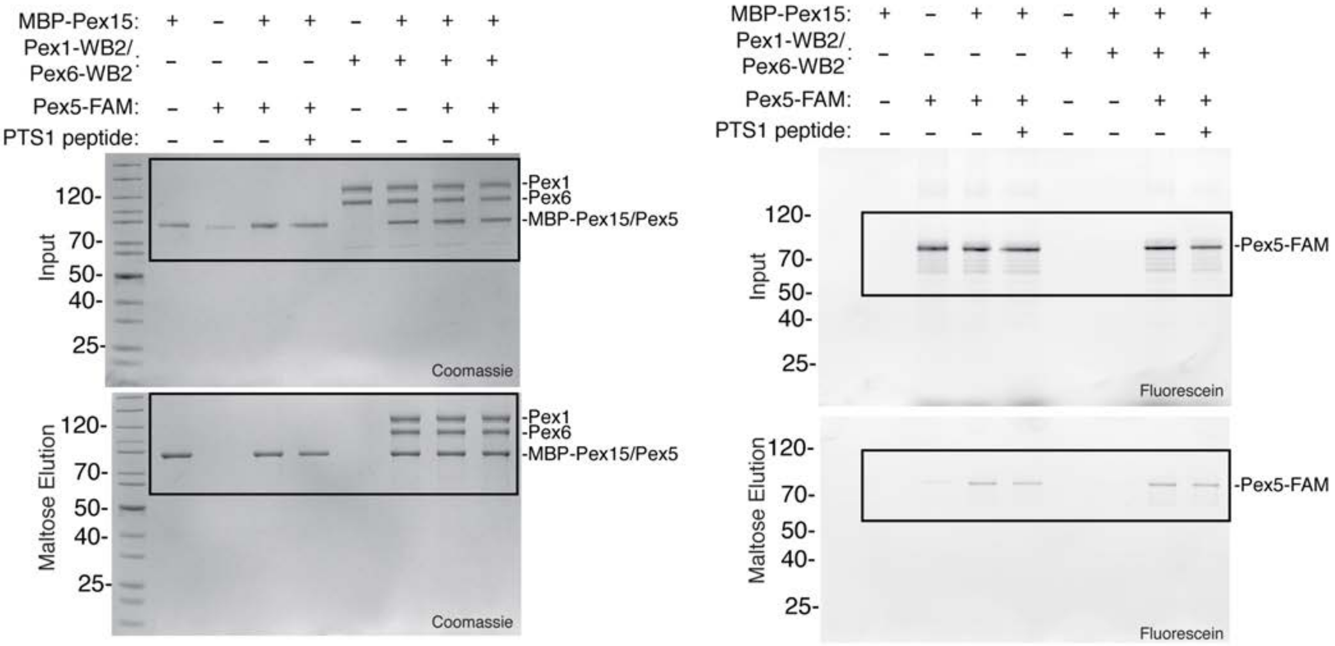

Uncropped blot for Supplementary Figure 4b.

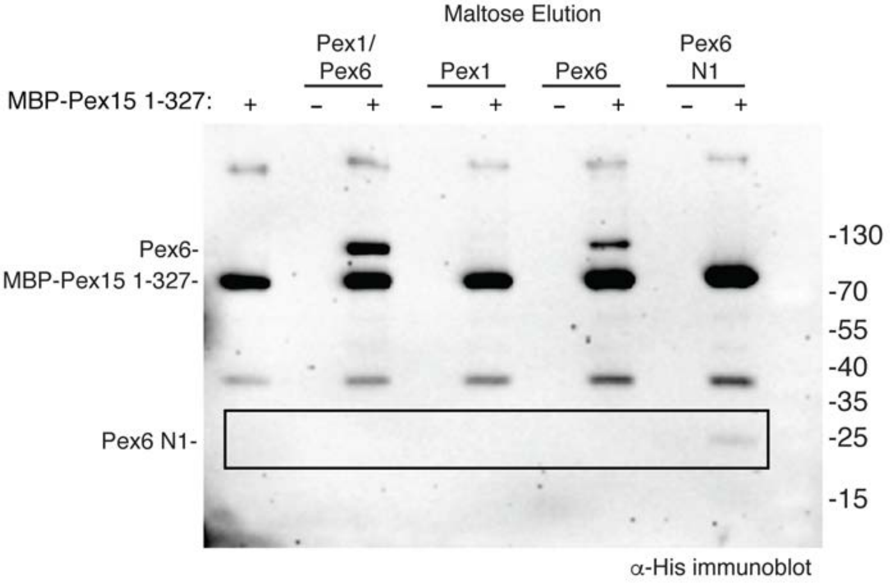

Uncropped gels for Supplementary Figure 8a.

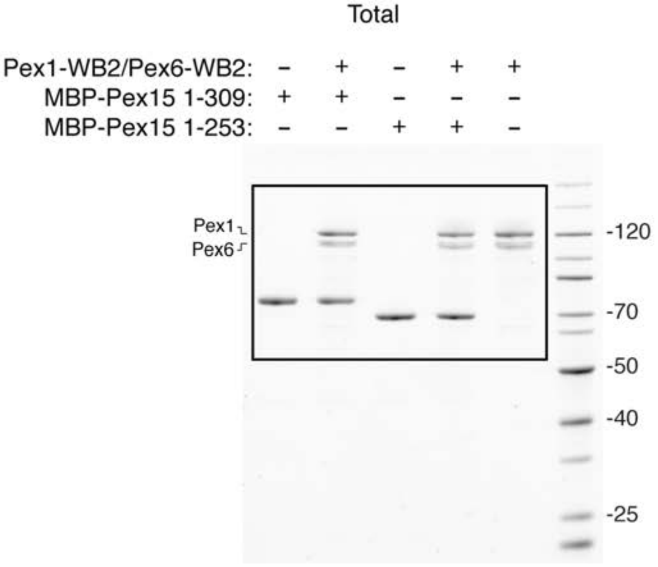

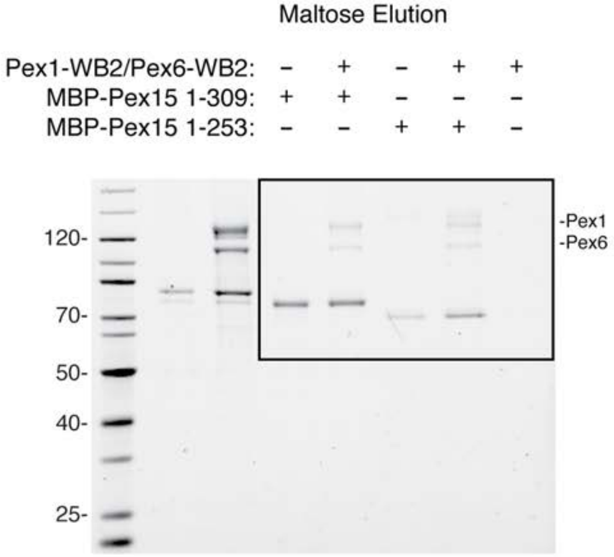

Uncropped gels for Supplementary Figure 8b.

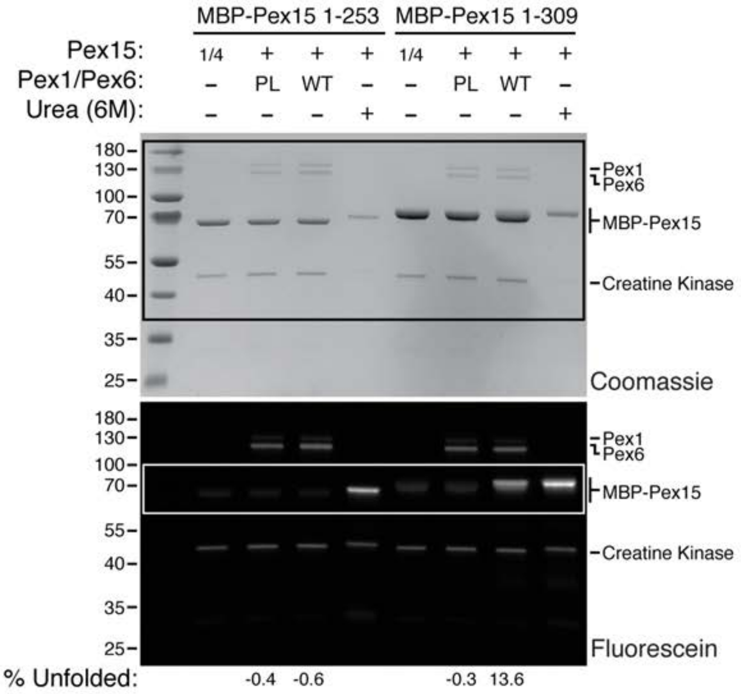

Uncropped gels for Supplementary Figure 8c.

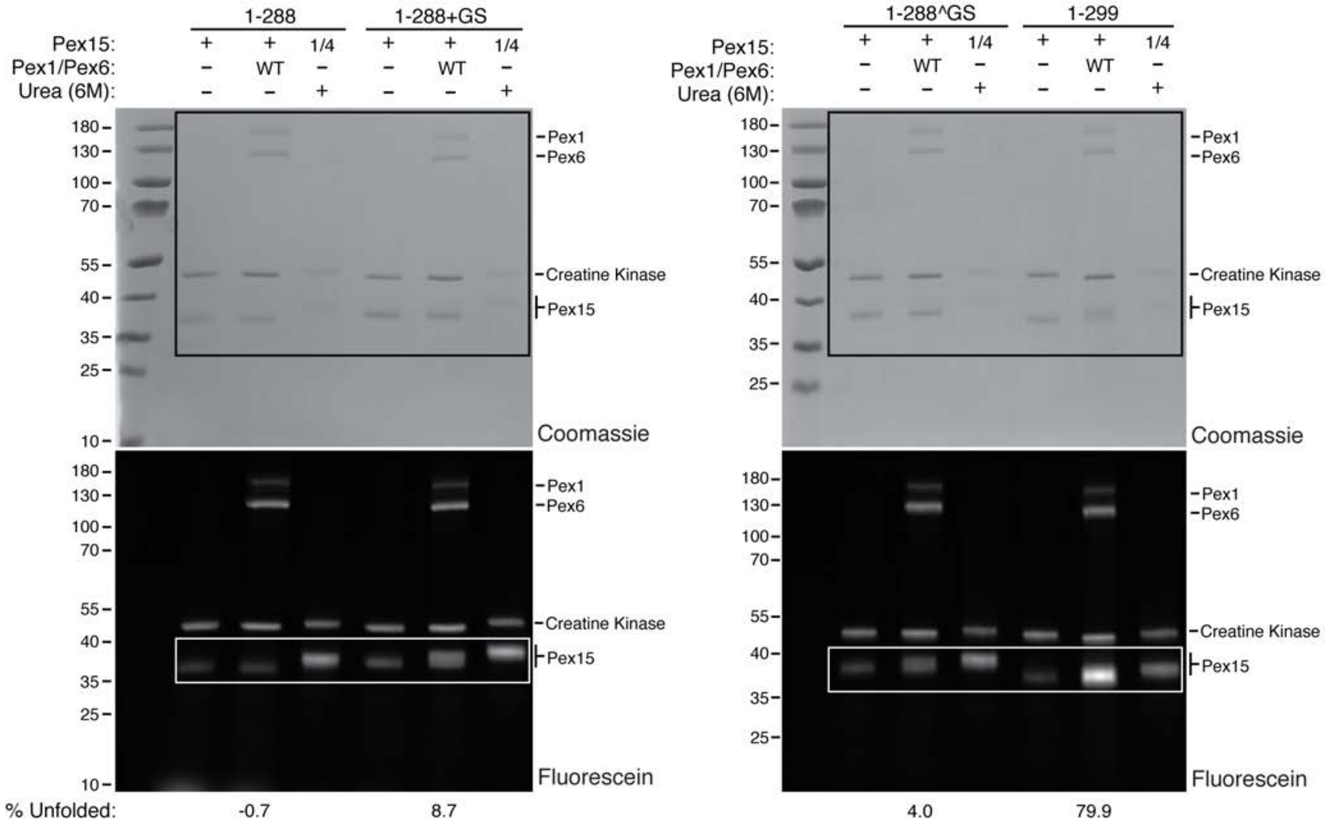

Supplementary Table 1

	$Pex15^{43-253}$
**Data collection**	
Wavelength (Å)	0.979560
Space group	P 21 21 21
Cell dimensions	
a, *b*, *c* (Å)	47.7, 58.8, 85.8
α, β, γ (°)	90.0, 90.0, 90.0
Resolution (Å)	48.52-1.55 (1.605-1.55)
R_{merge}	1.333 (1.486)
$I / \sigma I$	18.08 (1.80)
Completeness (%)	100 (100)
Redundancy	13.0 (12.4)
**Refinement**	
Resolution (Å)	48.52-1.55 (1.605-1.55)
No. reflections	35741 (3509)
R_{work} / R_{free}	0.1738 (0.2705) / 0.1890 (0.3022)
No. atoms	2035
Protein	1773
Water	262
B-factors	
Protein	20.71
Water	32.25
R.m.s. deviations	
Bond lengths (Å)	0.003
Bond angles (°)	0.56

*Dataset collected from one crystal. *Values in parentheses are for highest-resolution shell.

Supplementary Table 1. Data collection and refinement statistics for the crystal structure of the Pex15 core domain (SAD SeMet).

Supplementary Table 2

Sample	Deuteration (sec)	Calculated Mass of Undigested Fragment (Da)
Pex1-WB/Pex6-WB	0	21978.2
Pex1-WB/Pex6-WB	15	22027.3
Pex1-WB/Pex6-WB + Pex15	15	22021.2
Pex1-WB/Pex6-WB	600	22055.5
Pex1-WB/Pex6-WB + Pex15	600	22050.7

Supplementary Table 2. Pex15 protects an undigested fragment similar in mass to the Pex6 N1 domain. The calculated mass (Da) of the undigested fragment detected in samples with Pex1/Pex6 in the presence and absence of Pex15. The non-deuterated mass closely corresponds to the 21979 Da mass of the His-Pex6 aa 2-182, for which no peptides were observed (Supplementary Figure 3A). In the presence of Pex15, the undigested fragment is more protected, as observed by the decrease in the calculated molecular weight.

Supplementary Table 3

Pex15 Construct	Apparent K_D ± s.d. (μM)	Inhibited ATPase rate ± s.d. (% WT)
1-309	0.58 ± 0.12	18.1 ± 3.4
12-309	0.86 ± 0.12	16.2 ± 2.5
30-309	5.3 ± 0.81	22.0 ± 4.0
43-309	5.6 ± 0.50	18.6 ± 2.5
57-309	no fit	no fit
1-309 L22A L23A	8.7 ± 2.4	15.0 ± 9.8

Supplementary Table 3. Fit parameters for the Pex1/Pex6 ATPase inhibition by Pex15 variants.

Supplementary Table 4.

Construct	Primers (5'-3')	
Pex15 1-309	oDC80	oDC81
	TTTTTTACGGGGCGCCGTTC	CTGGAAGTCCTGTTCCAAGGTCCGggcagcagcG ATTATAAGGATGAC
Pex15 1-253	oDC82	oDC83
	CTGGAAGTCCTGTTCCAAGGT	CTTATCTTTTTGTATTTTATTATCTATTGAGTTCA GAATA
Pex15 43-253	oDC117	oDC118
	CATggtatatctccttcttaaagtta	AGTGAAGTATTTCAAGAATGTGT
Pex6 1-215	oDC157	oDC158a
	ACTAATTGTGCAGGGGAGAGA	TAAttaacctaggctgctgc
Pex15 1-299	oMS 001b	oMS 002b
	CTGGAAGTCCTGTTCCAAGGTC	GAGTCTACTATCAGTAGAGGTGCGT
Pex15 1-288	oMS 001b	oMS 003b
	CTGGAAGTCCTGTTCCAAGGTC	CGCATCCTCAACCTTAACCTG
Pex15 1-277	oMS 001b	oMS 004b
	CTGGAAGTCCTGTTCCAAGGTC	ATTGTGTAACATAGGCTCGTGAAAA
Pex15 1-266	oMS 001b	oMS 005b
	CTGGAAGTCCTGTTCCAAGGTC	TTTCTGTTTCACTCCTCTTTGTTTACC
Pex15 1-288^10GS	oMS 010	oDC247
	acttccgctgctaccATTGTGTAACATAGGCTCG	agcgggagtagcggtAGTAGTGAAGAACAGGTTAAGG
Pex15 1-288+11GS	oDC248	oDC249
	gcgggagtagcggtagtCTGGAAGTCCTGTTCCAAG	tacttccgctgctaccCGCATCCTCAACCTTAAC
Pex6 VT:AA	oDC267	oDC268
	ATCCAGGCGCAGTAGAGT	GCGGCGAGTGGATCGTTTATAACAGTGTCA
Pex1-F771A	Pex1 F771A for	Pex1 F771A rev
	AACAAGgctATAGGTGCCAGCGAACAAAACATAAG	TAAAATCTCTGGTCCCTTAACGGAGATAAAGTTTAAC
Pex6-Y805A	Pex6 Y805A for	Pex6 Y805A rev
	AATATGgcaATTGGTGAGAGTGAAGCTAATGTGC	CAACAGTTCAGGGCCTTTAACACTAAAAAAATT TAAAG
Pex1-E798Q	Pex1 B2 EQ forward	Pex1 B2 EQ reverse
	ATTCTATTGCGCCAAAGAGAGG	CGAAtTgGTCAAAAAATAGAATACAGGGTTTGAC
Pex6-E832Q	Pex6 B2 EQ forward	Pex6 B2 EQ reverse
	TGTCATATTTTTCGATcAgATCGattcagtagcacccaaac	CGATcTgATCGAAAAATATGACACAaggtttag
Pex1-K774S	Pex1 A2 KtoS	Pex1 A2 KtoSrev
	GGTtcAACGCTTCTGGCGAGCGCCGTG	ACAACCAGGATAACCGTAAAGCAAGATTC
Pex6-K778S	Pex6 A2 KtoS	Pex6 A2 KtoSrev
	GGTtcaACTCTAATGGCTAAGGCCATTGCAACAA ATTTTTC	TGTACCCGGTGGACCATAAAAAAGAATACCG

Construct	Primers (5'-3')	
Pex1-R852K	Pex1 R852K f	Pex1 Rfinger ATH
	GTTAaagCCGGGAAGATTAGACaaaagtg	AATGCGCTATCAATCAAATCAGG
Pex6-R889K	Pex6 R889K f	Pex6 Rfinger ATH
	CTACTAaagCCAGGACGATTCGATAAATTG	TGCTTCGTCCAATAAGTCTGG
Pex1 in pRS316	Pex1 proF NotI	Pex1 termR SalI
	ttttGCGGCCGCgtgcaaccatttcaggctttc	tttGTCGACcttctttgtatagacttctgcgag
Pex6 in pRS316	Pex6 proF NotI	Pex6 termR SalI
	ttttGCGGCCGCaagaactttatatatcatgtagcc	tttGTCGACgtataatcaactactagtatcttttctg
Pex1- *E. coli* to Yeast	Pex1 start reverse, for Gibson backbone	Pex1 term forward, for Gibson backbone
	GGATTCTCAGATTCTCAAACTTCAACC	GTGAGAATTCCATCGACATTGGTAGC
	ATH Pex1Start	Pex1 term reverse, for Pex1 Gibson
	ATGACGACGACCAAGAGGTTG	TCACATAAGGGAGAGTCGGCTACCAATG
Pex6- *E. coli* to Yeast	Pex6 start reverse, for Gibson backbone	Pex6 term forward, for Gibson backbone
	GCGTATATTCCGGAGAGACTAAACG	GAATCATTATGAAGCGGTGAGAGC
	Pex6Start	Pex6 term reverse, for Pex6 Gibson
	ATGAAGGCATCGCTTACGTTTAG	TTAAGCACCTTCAAAATTAGCTCTCACC
Pex5	Pex5 BamHI Start	Pex5 PacI End
	ttttGGATCCgATGGACGTAGGAAGTTGCTC	aaaattaattaaTCAAAACGAAAATTCTCCTTTAAATCTTTTC
Ub-Pex5-FLAG	Gibson Pex5-FLAG	Gibson Pex5-FLAG
	GCTAAGGCTAAGAGGTGGTATGGACGTAGGAAGTTGCTC	ggatcctcaTCATTATCATTTATCATCATCATCTTTATAATCAAACGAAAATTC
	Gibson pET28A Ub backbone	Gibson pET28A Ub backbone
	GAGCAACTTCCTACGTCCATACCACCTCTTAGCCTTAGC	GATGATGATGATAAATGATAATGAtgaggatccgaattcgag
Add FLAG to Pex5	FLAG-Pex5-rev	Pex5-D2-For
	CATCTTGTCATCGTCATCCTTATAATCCATggtatatctccttattaaagttaaacaaaattatttc	GACGTAGGAAGTTGCTCAGTGG
MBP-Pex15	BamHI pex15	T7term
	ttttGGATCCATGGCTGCAAGTGAGATAATG	ccgctgagcaataactagc
Pex15 12-309	Pex15 M12 forward	ATH remove UP
	ATGCATTCCCTCGATTCTTCTC	ggtatatctccttattaaagttaaacaaaattatttc
Pex15 30-309	Pex15 E30 forward	ATH remove UP
	ATGGAAAGTGATGAATCGACCAAGTC	ggtatatctccttattaaagttaaacaaaattatttc
Pex15 43-309	Pex15 S43 forward	ATH remove UP
	ATGAGTGAAGTATTTCAAGAATGTGTGAACC	ggtatatctccttattaaagttaaacaaaattatttc
Pex15 57-309	Pex15 D57 forward	ATH remove UP
	atgGATATCAAGGATTGCCTAGAAAAAATGTC	ggtatatctccttattaaagttaaacaaaattatttc
Pex15 L22AL23A	L22A L23A in Pex15 ATH	L22A L23A in Pex15 ATH rev
	gcAgcTAACGATGACTTATTCATTGAAAGTGATGAATCG	ATCCCTTAGAGAAGAATCGAGGGAATG

Construct	Primers (5'-3')	
Pex15 in pRS316	Pex15 proF NotI	Pex15 term SalI
	ttttgcggccgcCCGTACTCTCCAAGATAAAGGGAAAG	tttgtcgacTCGCATTGGTTGGACACGGTTAC
Pex15 Δ11 in pRS316	Pex15 M12 forward	Pex15pr Reverse
	ATGCATTCCCTCGATTCTTCTC	TATTCACAGAACCCTCTTGTATAAACC
Pex15 Δ29 in pRS316	Pex15 E30 forward	Pex15pr Reverse
	ATGGAAAGTGATGAATCGACCAAGTC	TATTCACAGAACCCTCTTGTATAAACC
Pex15 Δ42 in pRS316	Pex15 S43 forward	Pex15pr Reverse
	ATGAGTGAAGTATTTCAAGAATGTGTGAACC	TATTCACAGAACCCTCTTGTATAAACC
Pex15 Δ56 in pRS316	Pex15 D57 forward	Pex15pr Reverse
	atgGATATCAAGGATTGCCTAGAAAAAATGTC	TATTCACAGAACCCTCTTGTATAAACC
Pex15 Δ254-329 in pRS316	Pex15 aa 253 rev	Pex15 TMD
	CTTATCTTTTTGTATTTTATTATCTATTGAGTT	AGAAGTGTGCTGAACAAAAACGGACTTC
GST-Pex14	oDC87	oDC84
	CTTACGATCTTTACTGACCACGTCACTCATGGG CCCCTGGAACAGAAC	AATCGGATCTGGAAGTTCTGTTCCAGGGGCCC ATGAGTGACGTGGTCAGTAAAGATC
	oDC86	oDC85
	CATCATCACCACAGCCAGGATCCGATGTCCCC TATACTAGGTTATTGGAAAATTAAG	CCCTTAATTTTCCAATAACCTAGTATAGGGGACA TCGGATCCTGGCTGTGGTG
Add PP-His to Pex5	AddPrescissiontoPex5	AddHistagtoPex5
	CGGCCCCTGGAACAGAACTTCCAGcgacccAAA CGAAAATTCTCCTTTAAATCTTTTC	catcaccatcatcaccacTGAttaattaacctaggctgctgcc
MBP-Pex15 in pRS316	Gibson Pex15 promoter-MBP	Gibson MBP-Pex15 M1
	GGTGTGGTTTATACAAGAGGGTTCTGTGAATAat ggaagaaggtaaactggtaatctgg	TGTTCATTATCTCACTTGCAGCCATgctcgaattagtc tgcgcgtctttcagggc
	Pex15 M1	Pex15pr Reverse
	ATGGCTGCAAGTGAGATAATGAAC	TATTCACAGAACCCTCTTGTATAAACC
MBP-Pex15 1-253	Pex15 aa 253 rev	PP-FLAG
	CTTATCTTTTTGTATTTTATTATCTATTGAGTT	TTAGAGGTGCTATTCCAAGGTCCGGATTATAAG GATGACGATGACAAGc
MBP-Pex15 1-309	Pex15 aa 309 rev	PP-FLAG
	TTTTTTACGGGGCGCCGTTCCAG	TTAGAGGTGCTATTCCAAGGTCCGGATTATAAG GATGACGATGACAAGc
Replace FLAG with Strep (Pex1)	Pex1-Strep	STOP pCOLA ATH
	CTTCTCAAATTGAGGATGCGACCAgctgctgccCAT AAGGGAGAG	TGAttaattaacctaggctgctg

Construct	Primers (5'-3')	
Replace PP with Sortase (Pex5)	oDC141	pDC142
	CTGCCGGAAACCGGCGGCcatcaccatcatcaccacTGAt	cgacccAAACGAAAATTCTCC
Δpex15::kanMX	Pex15 5' UTR-F1	Pex15 3'UTR-R1
	CGTACTACGGTGTGGTTTATACAAGAGGGTTCTGTGAATAcggatccccgggttaattaa	GTCATTAAAATAAGTAGGTAGGGTTTTATAAACTATTCAAATATTTCgaattcgagctcgtttaaac
Δpex1::NatMX	Pex1 5'UTR-F1	Pex1 3'UTR-R1
	TCGAATTTCTTCTGGAAGGGACGGCAGTAACAAGAAACACCTGAGGAACTGCTCTTTCAAcggatccccgggttaattaa	GCGCCGCATTTTTTGCCCTTTAAAGGGAAACGCGCTTTGTTCTTTTCTTCTTCCTTTTCAgaattcgagctcgtttaaac
Δpex6::NatMX	Pex6 5'UTR-F1	Pex6 3'UTR-R1
	GTGGAGAGCATATATGTGTTTGCATACCCTCCAAAAGAAAGCGATTATAGTAACATTAATcggatccccgggttaattaa	GGCAAAATATGGGACATATATTTACAAATTTACCTATACGCTCTGAGTTGATATTACTTAgaattcgagctcgtttaaac

Supplementary Table 4. Primer sequences.
